# Defining the hydrophobic interactions that drive competence stimulating peptide (CSP)-ComD binding in *Streptococcus pneumoniae*

**DOI:** 10.3762/bjoc.14.151

**Published:** 2018-07-16

**Authors:** Bimal Koirala, Robert A Hillman, Erin K Tiwold, Michael A Bertucci, Yftah Tal-Gan

**Affiliations:** 1 Department of Chemistry, University of Nevada, Reno, 1664 North Virginia Street, Reno, Nevada, 89557, United States; 2 Department of Chemistry, Moravian College, 1200 Main Street, Bethlehem, Pennsylvania, 18018, United States

**Keywords:** binding surface, competence stimulating peptide (CSP), protein–peptide interactions, quorum sensing, *Streptococcus pneumoniae*, structure–activity relationships (SAR)

## Abstract

Quorum sensing (QS) is a cell–cell communication mechanism that enables bacteria to assess their population density and alter their behavior upon reaching high cell number. Many bacterial pathogens utilize QS to initiate an attack on their host, thus QS has attracted significant attention as a potential antivirulence alternative to traditional antibiotics. *Streptococcus pneumoniae*, a notorious human pathogen responsible for a variety of acute and chronic infections, utilizes the competence regulon and its associated signaling peptide, the competence stimulating peptide (CSP), to acquire antibiotic resistance and establish an infection. In this work, we sought to define the binding pockets within the ComD1 receptor used for binding the hydrophobic side-chain residues in CSP1 through the introduction of highly-conservative point mutations within the peptide. Optimization of these binding interactions could lead to the development of highly potent CSP-based QS modulators while the inclusion of non-natural amino acids within the CSP sequence would confer resistance to protease degradation, a requirement for drug candidates.

## Introduction

Quorum sensing (QS), a cell-density mechanism utilized by bacteria to assess their population density through the detection of diffusible signal molecules, enables bacterial species to synchronize their behavior and work as a multi-cellular organism at high cell numbers to achieve transformations that require population-wide efforts [1–2]. Many symbiotic and pathogenic phenotypes are regulated by QS, including bioluminescence, root nodulation, sporulation, swarming, biofilm formation, virulence factor production and competence [3–5]. As such, QS has attracted significant attention as a means to control bacterial behaviors (i.e., promote productive processes while attenuating harmful traits). Extensive work aimed at developing small molecule-based QS modulators against a multitude of Gram-negative bacterial species, including *Pseudomonas aeruginosa*, *Vibrio fischeri*, *Vibrio harveyi*, *Vibrio cholerae*, and *Acinetobacter baumannii* has been conducted [6–10]. Contrary, with the exception of the accessory gene regulator (agr) QS circuitry in *Staphylococcus aureus* [11–16], Gram-positive QS systems are underrepresented in the literature. To address this issue, our research groups have been actively working to delineate the molecular mechanisms of several Gram-positive QS circuitries, including *Enterococcus faecalis* [17], *Streptococcus gallolyticus* subsp. *gallolyticus* [18], *Streptococcus pneumoniae* [19], and *Lactobacillus plantarum*. These circuitries are usually centered on a peptide signal, rather than a small molecule, and are fruitful ground for the development of peptide-based therapeutics.

**Figure 1:**
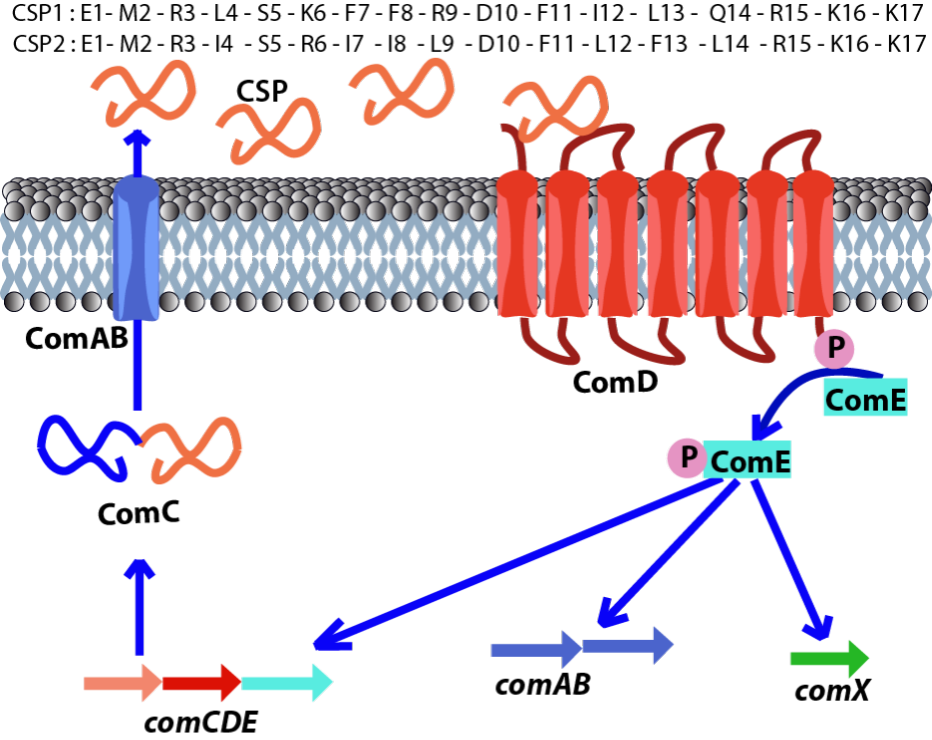
The competence regulon QS circuitry in *S. pneumoniae*. ComC is processed and secreted by ComAB as the mature CSP signal. Upon reaching a threshold concentration, CSP binds and activates the transmembrane histidine kinase receptor ComD. Upon activation, ComD phosphorylates ComE. Phosphorylated ComE then autoactivates the competence QS circuit and upregulates the expression of genes involved in virulence factor production, biofilm formation and attaining genetic competence through ComX. The sequences of the two CSP signals identified in *S. pneumoniae* are shown at the top.

*S. pneumoniae* is an opportunistic human pathogen that is responsible for a variety of acute and chronic infections, including pneumonia, bacteremia, sepsis, meningitis and otitis media, resulting in >22,000 deaths and direct medical costs totaling $3.5 billion a year in the United States alone [20–21]. The QS circuitry of *S. pneumoniae,* known as the competence regulon, is centered on the competence stimulating peptide (CSP, Figure 1). *S. pneumoniae* utilizes the regulon to become competent and acquire antibiotic resistance from the environment, initiate its attack on the human host through virulence factor production, and protect itself from the environment by forming biofilms [22–27]. The competence regulon in *S. pneumoniae* is therefore a major regulator of pathogenicity and thus a potential target for attenuating *S. pneumoniae* infections. *S. pneumoniae* strains can be divided into two main specificity groups based on the CSP signal that they produce (CSP1 and CSP2, Figure 1) and their cognate receptors (ComD1 and ComD2, respectively), with minimal cross-talk between the groups [28]. The two CSP signals share approximately 50% homology and differ mainly in hydrophobic residues in the central region of the peptides, suggesting that these residues are involved in receptor binding and specificity [29].

Previously, Yang et al. conducted a systematic structure–activity relationship (SAR) analysis of the CSP1 scaffold and found that the hydrophobic residues in positions 4, 7, 8, 11, 12 and 13, along with Arg3, are important for ComD1 binding [19]. Moreover, three of these positions, 4, 7 and 8, were suggested by Johnsborg et al. to confer specificity between the ComD receptors [29]. Therefore, in this work, we aimed to define the hydrophobic pockets within the ComD1 receptor that are occupied by the hydrophobic residues in positions 4, 7, 8, 11, 12 and 13 as a means to enhance the binding interactions between CSP1 and ComD1. To this end, we utilized highly conservative mutations in these positions using both proteogenic and non-proteogenic amino acids and assessed the effects of these mutations on both receptor binding and specificity. Our analysis revealed that positions 4, 7, 8 and 11 are more resistant to modification than positions 12 and 13. Furthermore, it appears from our analysis that the side-chain residues do not occupy 100% of the binding pockets, thus these pockets can accommodate better elongated side-chain residues compared to truncated side-chains or those that introduce electrostatic effects. Finally, our results further correlated helicity with bioactivity. Combined, the results of this study can be used to design novel CSP-based QS modulators with improved pharmacological properties that could be applied to study QS in vivo.

## Results and Discussion

### Design and synthesis of CSP1 analogs

**Figure 2:**
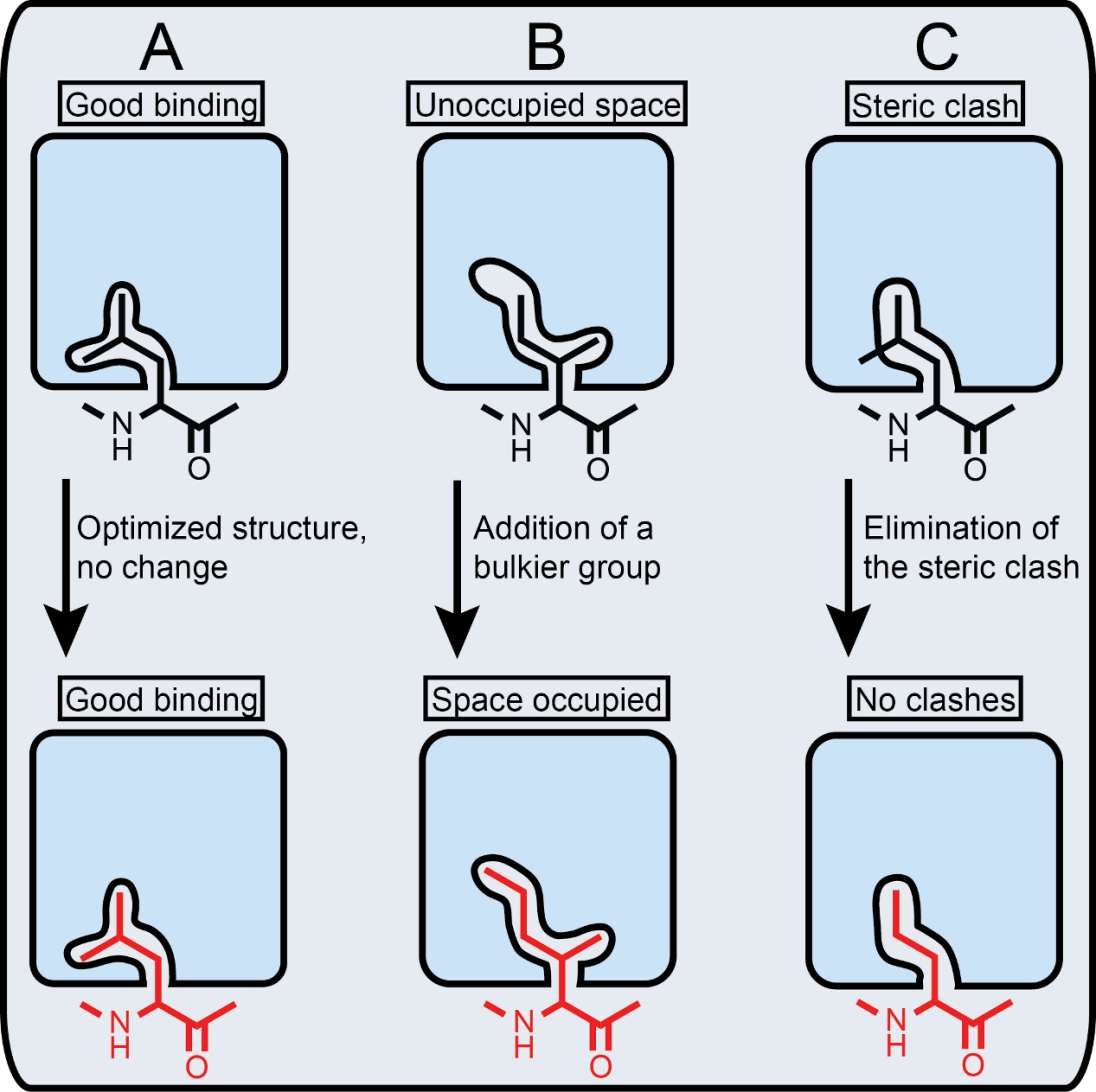
Assessment of protein–peptide binding pockets. Key side-chain residues (black) can either fully occupy their binding pocket (A), partially occupy their binding pocket (B), or have some unfavorable steric clashes (C). As such, no optimization (red) is required in (A), larger bulkier side-chain can be introduced to improve binding interactions in (B), while smaller side-chain residue can be introduced in (C) to eliminate steric clashes and improve binding.

**Figure 3:**
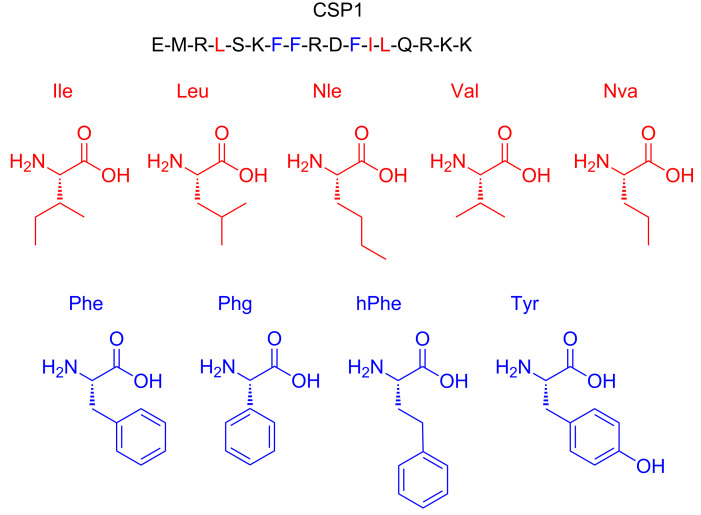
Design of CSP1 analogs. The CSP1 sequence is presented using the one-letter amino acid code. Residues in red were replaced by all the other red residues, while residues in blue were replaced by all the other blue residues. Nle, norleucine; Nva, norvaline; Phg, phenylglycine; hPhe, homophenylalanine.

In this work, we aimed to define the binding pockets in ComD1 that accommodate the hydrophobic side-chain residues in CSP1 and determine their degree of occupancy as a means to optimize CSP1–ComD1 interactions and develop novel CSP-based QS modulators with improved activities. When optimizing protein–peptide interactions, it is important to determine which key side-chain residues within the peptide sequence fully occupy their binding pocket within the protein and which ones do not optimally occupy their binding site, either by not occupying the entire binding pockets or by having some unfavorable steric clashes (Figure 2). To do so, one can either use computational models, when structural information of the protein/receptor is available [30], or utilize conservative point mutations within the ligand peptide to assess the occupancy level and degree of specificity. Since no structural information is available for the ComD receptors, we chose to assess the ComD1 binding pocket by synthesizing a set of CSP1 analogs bearing highly conservative point mutation in key hydrophobic positions (4, 7, 8, 11, 12 and 13). Aliphatic hydrophobic side-chains, namely Leu or Ile, were replaced with proteogenic and non-proteogenic aliphatic residues (Ile, Leu, Val, norleucine (Nle), or norvaline (Nva)), while aromatic hydrophobic residues (Phe) were replaced with other aromatic residues (phenylglycine (Phg), homophenylalanine (hPhe), or Tyr; Figure 3). The CSP1 analogs were constructed using standard solid-phase peptide synthesis (SPPS) protocols (see Materials and Methods for SPPS procedures), followed by purification to homogeneity by semipreparative RP-HPLC (see the Supporting Information File 1 for full characterization details).

### Structure–activity relationships of CSP1 analogs

To assess QS modulation, we utilized the β-gal reporter strains, constructed by Lau and co-worker [31]. In these strains the *lacZ* gene is under the control of QS (pcomX). Thus, upon QS activation, ComE will bind pcomX and transcribe *lacZ* (in addition to upregulation of ComX). ComD modulation can therefore be quantified by measuring β-gal activity. The peptides were first screened for their ability to activate/inhibit the ComD1 and ComD2 receptors at high concentration (10 µM). Only analogs that exhibited greater than 75% activation compared to the native signal (CSP1 or CSP2) or greater than 50% inhibition of the maximal signal induced by the native peptide were further evaluated to determine their EC_50_/IC_50_ values, respectively.

Starting with the ComD1 receptor, generally, it appears that the binding sites of the CSP1 aromatic residues are optimally occupied, leading to a more significant reduction in potency when the side-chain residue is changed compared to the aliphatic binding pockets (Table 1). Looking at the aliphatic residues in CSP1, the binding pocket for the fourth residue, leucine, can accommodate elongation of the aliphatic chain by one methylene (Leu → Nle) as well as movement or loss of chain-branching (Leu → Ile, Nle or Nva; Table 1). Contrary, truncation of the aliphatic chain by one carbon (Leu → Val) resulted in significant reduction in potency, suggesting that important binding interactions are occurring between the δ carbon of the Leu residue in CSP1 and the ComD1 binding pocket. Regarding the 12th position, Ile, it appears that the protein–peptide interface is more promiscuous, accommodating all types of modifications (Table 1). It seems that elongation by one carbon (Ile → Nle) as well as repositioning of branching (Ile → Leu) are slightly more tolerated than truncation (Ile → Val) or removal of branching (Ile → Nva). However, these changes were subtle (about 2-fold; Table 1). Overall, it appears from the results that the binding pocket of the 12th residue is not fully occupied by the side-chain residue and can be further optimized. Moving to the 13th position, Leu, this binding pocket also accommodated changes in carbon length and connectivity relatively well. In this case, relocation of branching (Leu → Ile) was most tolerated, followed by removal of branching, either with or without chain elongation (Leu → Nle or Nva; 2-fold reduction in potency). Truncation was least tolerable (Leu → Val; 3-fold decrease in potency). Combined, these results suggest that all three sites can be further optimized with an emphasis on utilizing longer, bulkier substituents. Moreover, the decreases in potency observed with the introduction of Val indicate that side chains containing δ carbons (at minimum) are required to preserve QS activity.

**Table 1:** Biological and structural characterization of the CSP1 analogs^a^.

Peptide name	ComD1		ComD2		Helicity (%)^d^
	EC_50_ (nM)^b^	95% CI^c^	EC_50_ (nM)	95% CI	

CSP1	10.3	6.27–16.8	526	498–556	20.1%
CSP1-L4I	10.2	6.74–15.4	>1000	–	34.8%
CSP1-L4NL	13.5	6.38–28.5	>1000	–	29.2%
CSP1-L4NV	5.74	2.94–11.2	627	332–1180	34.0%
CSP1-L4V	113	74.1–171	–^e^	–	30.1%
CSP1-F7FG	81.3	35.1–188	828	512–1340	26.3%
CSP1-F7HF	81.7	61.8–108	317	148–682	26.7%
CSP1-F7Y	344	155–764	–^e^	–	31.8%
CSP1-F8FG	884	514–1520	–^e^	–	13.4%
CSP1-F8HF	43.4	35.1–53.5	>1000	–	19.4%
CSP1-F8Y	85.0	72.2–100	–^e^	–	25.7%
CSP1-F11FG	>1000	–	>1000	–	15.9%
CSP1-F11HF	65.8	41.8–104	>1000	–	32.3%
CSP1-F11Y	95.6	59.4–154	–^e^		29.2%
CSP1-I12L	8.56	5.42–13.5	537	384–752	34.1%
CSP1-I12NL	6.68	3.52–12.6	>1000	–	24.7%
CSP1-I12NV	13.8	11.8–16.2	853	748–973	25.6%
CSP1-I12V	15.3	6.72–34.9	–^e^	–	29.0%
CSP1-L13I	9.12	5.96–13.9	705	426–1170	27.7%
CSP1-L13NL	18.6	7.95–43.3	>1000	–	17.3%
CSP1-L13NV	15.2	7.56–30.3	>1000	–	30.9%
CSP1-L13V	31.0	20.8–46.1	–^e^	–	25.8%

^a^See experimental section for details of reporter strains and methods. See Supporting Information File 1 for plots of agonism dose response curves and CD spectra. All bioassays were performed in triplicate. ^b^EC_50_ values determined by testing peptides over a range of concentrations. ^c^95% confidence interval. ^d^Percent helicity determined from CD spectra in 20% TFE using the absorbance at 222 nm [32]. ^e^EC_50_ not determined due to the analog’s low induction in primary agonism screening assay.

Turning to the aromatic residues, all of which are Phe, repositioning of the benzene ring at the seventh position (truncation or elongation, Phe → Phg or hPhe, respectively) led to an 8-fold reduction in potency. These results suggest that this ring sits in a relatively tight pocket (Table 1). Moreover, addition of a hydroxy group (Phe → Tyr) resulted in a 33-fold reduction in potency, providing further support regarding the specificity of the binding pocket, specifically with regards to electronic/polar effects. The eighth position exhibited an interesting trend where truncation of the side-chain (Phe → Phg) was not tolerated (>80-fold reduction in potency) while elongation of the chain (Phe → hPhe) resulted in only a modest reduction in potency (4-fold change; Table 1). In this case, even the addition of a polar hydroxy moiety (Phe → Tyr) was relatively tolerated, resulting in an 8-fold reduction in potency. Together, these results suggest that the binding pocket for the eighth residue is not as optimally occupied as the one for the seventh residue. An identical trend to the 8th residue was also observed for the 11th residue, with only modest variations in potencies (Table 1). Combined, these results suggest that the binding pockets for the aromatic residues are mostly occupied and the benzene ring must be far enough from the CSP backbone in order to maintain helicity (see Structural Analysis below) and effectively interact with the binding pocket. Thus, pending no unexpected enhancement from isosteric substitutions (e.g., pyridylalanine, cyclohexylalanine, etc.), they are likely not ideal positions for further optimization.

With regards to the ComD2 receptor, since we performed highly-conservative mutations to the CSP1 scaffold, we did not expect significant changes in potencies against the ComD2 receptor, compared to CSP1. Indeed, most of the analogs exhibited similar activities to CSP1 against ComD2 (Table 1; <2-fold change). Interestingly, two mutations were not tolerable and resulted in significant loss of activity: These were Phe → Tyr for positions 7, 8 and 11, as well as Leu/Ile → Val for positions 4, 12 and 13. The valine substitution results are in agreement with the trend observed for the ComD1 receptor and further highlight the importance of the chain-length for effective binding, while the tyrosine substitution results suggest that the binding pockets within the ComD2 receptor cannot accommodate polar/electron-rich substituents.

### Structural analysis of CSP1 analogs

Next, we wanted to assess the impact our modifications to the CSP1 scaffold had on its conformation. We utilized circular dichroism (CD) spectroscopy to evaluate the main structural motifs of the different analogs. Since only conserved modifications were introduced to the CSP1 sequence, we did not expect significant changes to the overall structural characteristics. Indeed, all the analogs exhibited α-helix CD spectra in membrane mimicking conditions (20% trifluoroethanol (TFE) in PBS buffer; Figure S4, Supporting Information File 1). Quantification of the helix content using both the mean residue ellipticity at 222 nm [32] and the BeStSel method [33] yielded similar trends (Table S2, Supporting Information File 1). Importantly, the two analogs that exhibited the lowest percent helicity, CSP1-F8FG and CSP1-F11FG, were the least active analogs against the ComD1 receptor (Table 1), supporting the hypothesis that an α-helix is required for effective ComD1 binding.

**Figure 4:**
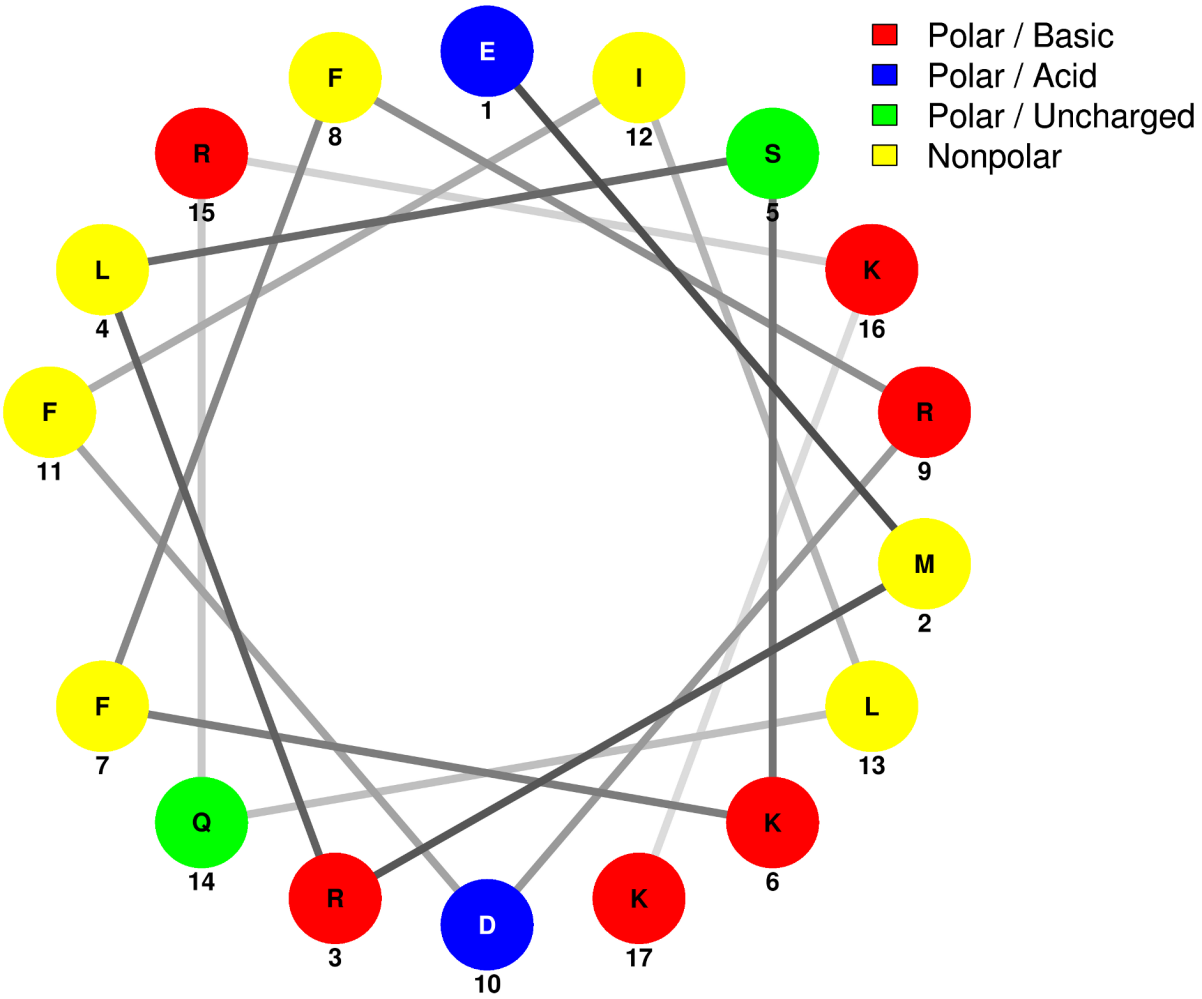
Helical wheel representation of CSP1. The CSP1 residues are presented using the one-letter amino acid code. The presentation reveals that, with the exception of L13, all the hydrophobic residues occupy the same face of the helix. Image produced using the NetWheels application (http://lbqp.unb.br/NetWheels/).

A helical wheel representation of the CSP1 sequence revealed that, with the exception of L13, all the residues discussed above (4, 7, 8, 11 and 12) occupy the same face of the helix (Figure 4). This result suggests that only one face of the CSP1 helix is directly interacting with the ComD1 receptor. Interestingly, L13 is predicted to be positioned on the opposite face of the helix, away from the proposed binding interface between CSP1 and ComD1. It is therefore not clear why this residue was found to be important for effective receptor binding. In-depth structural analysis of CSP1 in membrane mimicking conditions using NMR revealed that CSP1 adopts a kinked α-helix conformation, pointing the Leu13 side-chain more closely to the other hydrophobic side-chain residues than predicted by the helical wheel diagram (Yang et al. unpublished results). The kinked α-helix conformation may explain the importance of Leu13 in receptor binding. Alternatively, since the ComD receptors are predicted to dimerize upon activation, Leu13 may have a role in stabilizing the dimerization process prior to phosphorylating the response regulator, ComE. Lastly, it is possible that CSP1 interacts with the ComD1 receptor using more than just a single helical face. Additional structural studies are needed to test these hypotheses and conclusively determine the role of Leu13 in ComD1 binding and activation.

## Conclusion

In conclusion, we incorporated highly conservative point mutations to the CSP1 sequence in order to define the hydrophobic binding pockets within the ComD1 receptor. Our analysis revealed that the binding pockets of the 4th, 12th and 13th positions in CSP1 are likely not optimally occupied by the current side-chain residues and can thus be the focus for optimization in order to obtain more potent CSP-based QS modulators. Our analysis also revealed that the binding pockets of the aromatic side-chains in CSP1 are likely optimally occupied by the current residues (Phe) and should thus be retained to maintain high affinity to the ComD1 receptor. Lastly, structural analysis using CD spectroscopy of the CSP1 analogs provided additional support that an α-helix conformation is required for effective ComD1 binding by CSP1. We believe that the insights revealed in this study are valuable to the development of highly potent CSP-based QS modulators with enhanced pharmacological properties.

## Materials and Methods

**Chemical reagents and instrumentation.** All chemical reagents and solvents were purchased from Sigma-Aldrich and used without further purification. Water (18 MΩ) was puriﬁed using a Millipore Analyzer Feed System. Solid-phase resins were purchased from Chem-Impex or P3 Biosystems.

Reversed-phase high-performance liquid chromatography (RP-HPLC) was performed using two Shimadzu systems each equipped with a CBM-20A communications bus module, two LC-20AT pumps, an SIL-20A auto sampler, an SPD-20A UV–vis detector, a CTO-20A column oven, one with an FRC-10A fraction collector and one without. Matrix-assisted laser desorption ionization time-of-ﬂight mass spectrometry (MALDI–TOF MS) data were obtained on either a Bruker Autoflex or Bruker Microflex spectrometer equipped with a 60 Hz nitrogen laser and a reﬂectron. In positive ion mode, the acceleration voltage on Ion Source 1 was 19.01 kV. Exact mass (EM) data were obtained on an Agilent Technologies 6230 TOF LC/MS spectrometer. The samples were sprayed with a capillary voltage of 3500 V and the electrospray ionization (ESI) source parameters were as follows: gas temperature of 325 °C at a drying gas flow rate of 3 L/min at a pressure of 25 psi.

**Peptide synthesis.** All the CSP1 analogs were synthesized on a 4-benzyloxybenzyl alcohol (Wang) resin (0.65 mmol/g) pre-loaded with Fmoc-L-Lys(Boc). With the exception of the phenylglycine and norvaline derivatives, the CSP1 analogs were synthesized using standard Fmoc-based solid-phase peptide synthesis (SPPS) procedures [34]. Phenylglycine and norvaline derivatives were prepared on a CEM Discover microwave synthesizer, with diisopropyl carbodiimide (DIC) as the coupling reagent along with Oxyma Pure. The ratio of DIC:Oxyma Pure:AA was 3.6:3:3 dissolved in *N*,*N*-dimethylformamide (DMF) for a final DIC concentration of 0.2 M. Reactions were run at 50 W at a temperature of 75 °C for 8 minutes, followed by 2 × 3 min deprotection with 20% piperidine in DMF.

**Peptide purification.** Crude peptides were purified with RP-HPLC. The crude peptide was dissolved in ACN/H_2_O (1:4) and purified in 2.0–2.4 mL portions on either a Phenomenex Luna (5 µm, 10 mm, 150 mm, 100 Å) C18 column or a Phenomenex Kinetex (5 µm, 10 mm, 250 mm, 110 Å) C18 column with a flow rate of 5 mL/min; mobile phase A = 18 MΩ water + 0.1% TFA and mobile phase B = ACN + 0.1% TFA. The collected fraction was lyophilized overnight and dissolved again in ACN/H_2_O (1:4) for a second prep run. Preparative HPLC methods were used to separate the crude peptide mixture to different chemical components using a linear gradient (first prep 15% B → 45% B over 30 min and second prep 25% B → 35% B over 30 min) as described in [19]. Then, either an analytical Phenomenex Luna C18 column (5 µm, 4.6 mm, 150 mm, 100 Å) or an analytical Phenomenex Kinetex C18 column (5 µm, 4.6 mm, 250 mm, 110 Å) was used to quantify the purity of the desired fraction using a linear gradient (5% B → 95% B over 22 min or 27 min, respectively). Purities were determined by integration of peaks with UV detection at 220 nm. Only peptide fractions that were purified to homogeneity (>95%) were used for the biological assays. TOF-MS was used to validate the presence of synthesized peptides. The observed mass-to-charge (*m*/*z*) ratio of the peptide was compared to the expected *m*/*z* ratio for each peptide (see Table S1, Supporting Information File 1).

**Biological reagents and strain information.** All standard biological reagents were purchased from Sigma-Aldrich and used according to enclosed instructions. Donor horse serum (defibrinated) was purchased from Sigma-Aldrich and stored at 4 °C until use in bacterial growth conditions.

To examine the ability of the synthesized CSP analogs to modulate the ComD receptors, and thus the QS circuit in *S. pneumoniae*, β-galactosidase assays were performed using D39pcomX::lacZ (group I) and TIGR4pcomX::lacZ (group II) reporter strains as described in [19].

**Bacterial growth conditions.** Bacteria from a freezer stock were grown as described in [19]. Briefly, the bacteria were streaked into a THY agar plate supplemented with 5% donor horse serum and chloramphenicol at a final concentration of 4 µg/mL. The plate was incubated for 8 h in a CO_2_ incubator (37 °C with 5% CO_2_). Fresh colonies (single colony for D39pcomX::lacZ; multiple colonies for TIGR4pcomX::lacZ) were picked into sterilized cultural tubes containing 5 mL of THY broth supplemented with chloramphenicol at a final concentration of 4 µg/mL and the cultures were incubated in a CO_2_ incubator overnight (15 h). Overnight cultures were then diluted (1:50 for D39pcomX::lacZ; 1:10 for TIGR4pcomX::lacZ) with THY and the resulting solution was incubated in a CO_2_ incubator for 3–4 hours, until the bacteria reached early exponential stage (0.30–0.35 for D39pcomX::lacZ; 0.20–0.25 for TIGR4pcomX::lacZ) as determined by using a plate reader.

**β-Galactosidase assay. Activation assays.** The ability of synthetic CSP1 analogs to activate the expression of *comX* was determined using reporter strains grown in THY as described in [19]. Briefly, an initial activation screening was performed at high concentration (10 µM) for all CSP analogs. 2 µL of 1 mM solution of CSP analogs in dimethyl sulfoxide (DMSO) were added in triplicate to a clear 96-well microtiter plate. 2 µL of 20 µM solution of CSP1 were added in triplicate and served as the positive control for the group I strain (D39pcomX::lacZ), while 2 µL of 100 µM solution of CSP2 were added as the positive control for the group II strain (TIGR4pcomX::lacZ). These concentrations were chosen to afford full activation of the QS circuit, as determined from the dose-dependent curves created for the native CSPs. 2 µL of DMSO was added in triplicate and served as the negative control for both groups. Then, 198 µL of bacterial culture was added to each well containing CSP and analogs, the plate was incubated at 37 ºC for 30 minutes, and the OD_600nm_ was measured. In order to measure the β-galactosidase activity in the pneumococcal culture, the cells were lysed by incubating the culture for 30 minutes at 37 °C with 20 µL of 0.1% Triton X-100. In a new plate, 100 µL of Z-buffer solution (60.2 mM Na_2_HPO_4_, 45.8 mM NaH_2_PO_4_, 10 mM KCl, and 1.0 mM MgSO_4_ in 18 MΩ H_2_O; pH was adjusted to 7.0 and the buffer was sterilized before use) containing 2-nitrophenyl-β-D-galactopyranoside (ONPG) at a final concentration of 0.4 mg/mL was added, followed by 100 µL of lysate, and the plate was incubated for 3 hours at 37 ºC. The reaction was stopped by adding 50 µL of 1 M sodium carbonate solution, and the OD_420nm_ and OD_550nm_ were measured using a plate reader. The final results were reported as percent activation, which is the ratio between the Miller units of the analog and of the positive control. For calculation of Miller units, please see data analysis below. Analogs that exhibited high activity in the initial screening (see Figures S1 and S2, Supporting Information File 1) were further evaluated using a dose-dependent assay in which peptide stock solutions were diluted with DMSO in serial dilutions (either 1:2, 1:3, or 1:5) and assayed as described above. GraphPad Prism 5 was used to calculate the EC_50_ values, which are the concentration of a drug that gives half-maximal response.

**Inhibition assays.** Analogs that exhibited low *comX* activation in the initial screening (see Figure S2, Supporting Information File 1) were evaluated for competitive inhibition as described in [19]. Briefly, the ability of synthesized CSP analogs to inhibit the expression of *comX* by outcompeting CSP for the receptor binding site was evaluated using the same assay conditions as described above, except that in this case native CSP (for this purpose, CSP2) was added to every well in a set concentration (250 nM). This concentration was chosen to afford full activation of the QS circuit, as determined from the dose-dependent curves created for the native CSPs. 2 µL of a native CSP (25 µM solution) and 2 µL of a 1 mM solution of each CSP analog were added to the same well in triplicate in a clear 96-well microtiter plate. 2 µL of native CSP (25 µM solution) and 2 µL of DMSO were added to the same well in triplicate and served as the positive control. 4 µL of DMSO was added in triplicate and served as the negative control. Then, 196 µL of bacterial culture was added to the wells and the plate was incubated at 37 ºC for 30 minutes. The procedure for lysis, incubation with ONPG and all the measurements were as described in the activation assay. None of the analogs exhibited significant competitive inhibition in the initial screening (Figure S3, Supporting Information File 1).

**Analysis of activation/inhibition data.** Miller units were calculated using the following formula:


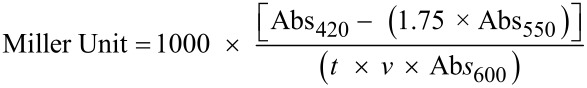


Abs_420_ is the absorbance of *ortho*-nitrophenol (ONP). Abs_550_ is the scatter from cell debris, which, when multiplied by 1.75 approximates the scatter observed at 420 nm. *t* is the duration of incubation with ONPG in minutes, *v* is volume of lysate in milliliters, and Abs_600_ reflects cell density.

**Circular dichroism (CD) spectroscopy.** CD spectra were recorded with an Aviv Biomedical CD spectrometer (model 202-01) as described in [19]. Briefly, all the measurements were performed with a peptide concentration of 200 µM in PBS buffer (137 mM NaCl, 2.7 mM KCl, 10 mM Na_2_HPO_4_, 1.8 mM KH_2_PO_4_; pH was adjusted to 7.4) with 20% trifluoroethanol (TFE). Measurements were performed at 25 ºC with a quartz cuvette (science outlet) with a path length of 0.1 cm. Samples were scanned one time at 3 nm min^−1^ with a bandwidth of 1 nm and a response time of 20 s over a wavelength range (195 to 260 nm). Percent helicity (*f*_H_) was calculated for all peptides using the following equation:


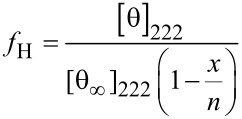


[θ]_222_ is the mean residue ellipticity of the sample peptide at 222 nm, [θ_∞_]_222_ is the mean residue ellipticity of an ideal peptide with 100% helicity (−44,000 deg cm^2^ dmol^−1^) [32], *n* is the number of residues in the potential helical region, and *x* is an empirical correction for end effects (2.5) [32]. Secondary structure contents were also calculated using the BeStSel (beta structure selection) method (http://bestsel.elte.hu/) [33].

## Supporting Information

File 1 Full details of peptide characterization, initial screening results, dose response curves for CSP1 analogs, and CD spectra of all the CSP1 analogs.
