# Supplementary material for: Defining the hydrophobic interactions that drive competence stimulating peptide (CSP)-ComD binding in Streptococcus pneumoniae
Source: Beilstein J Org Chem. 2018 Jul 16;14:1769–77. doi: 10.3762/bjoc.14.151 (PMC6071684; doi:10.3762/bjoc.14.151)
Supplement: File 1 — Full details of peptide characterization, initial screening results, dose response curves for CSP1 analogs, and CD spectra of all the CSP1 analogs. [file Beilstein_J_Org_Chem-14-1769-s001.pdf]

Supporting Information

for

Defining the hydrophobic interactions that drive competence stimulating peptide (CSP)-ComD binding in *Streptococcus pneumoniae*

Bimal Koirala[1], Robert A. Hillman[2], Erin K. Tiwold[2], Michael A. Bertucci*[2] and Yftah Tal-Gan*[1]

Address: [1]Department of Chemistry, University of Nevada, Reno, 1664 North Virginia Street, Reno, Nevada, 89557, United States and [2]Department of Chemistry, Moravian College, 1200 Main Street, Bethlehem, Pennsylvania, 18018, United States

[*] Corresponding author

Email: Michael A. Bertucci- bertuccim@moravian.edu; Yftah Tal-Gan - ytalgan@unr.edu

Full details of peptide characterization, initial screening results, dose response curves for CSP1 analogs, and CD spectra of all the CSP1 analogs

HPLC Traces for CSP1 analogs……………………………………………………………………..S2

MS and HPLC data for CSP1 analogs…………………………………………………………….S13

Bioassay initial screening results……………………………………………………………..S14

Dose response curves…………………………………………………………………………….S17

Circular dichroism (CD) spectra……………………………………………………………..S22

References……………………………………………………………………………………...S24

HPLC Traces for CSP1 analogs

CSP1-L4I

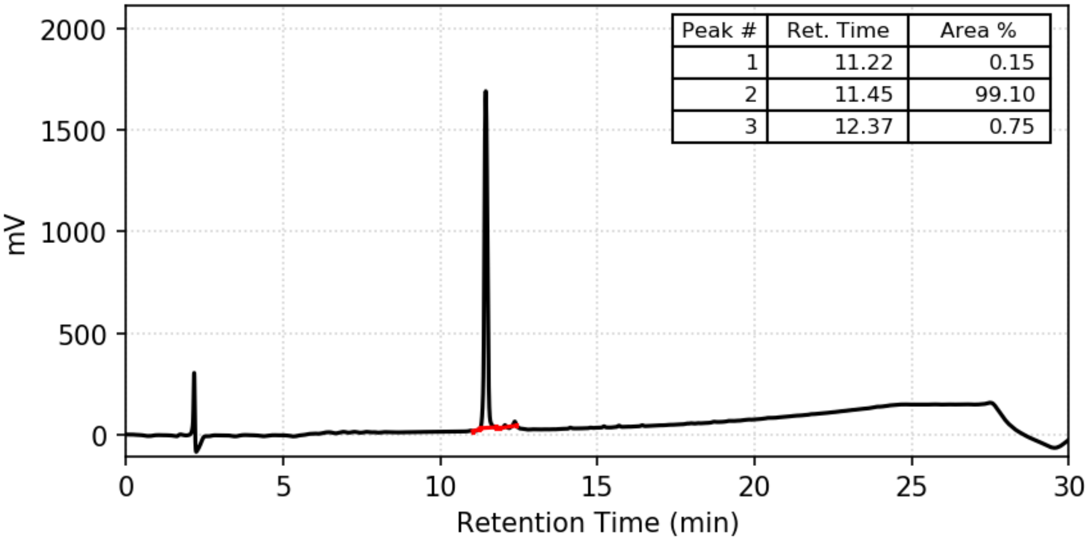

CSP1-L4NL

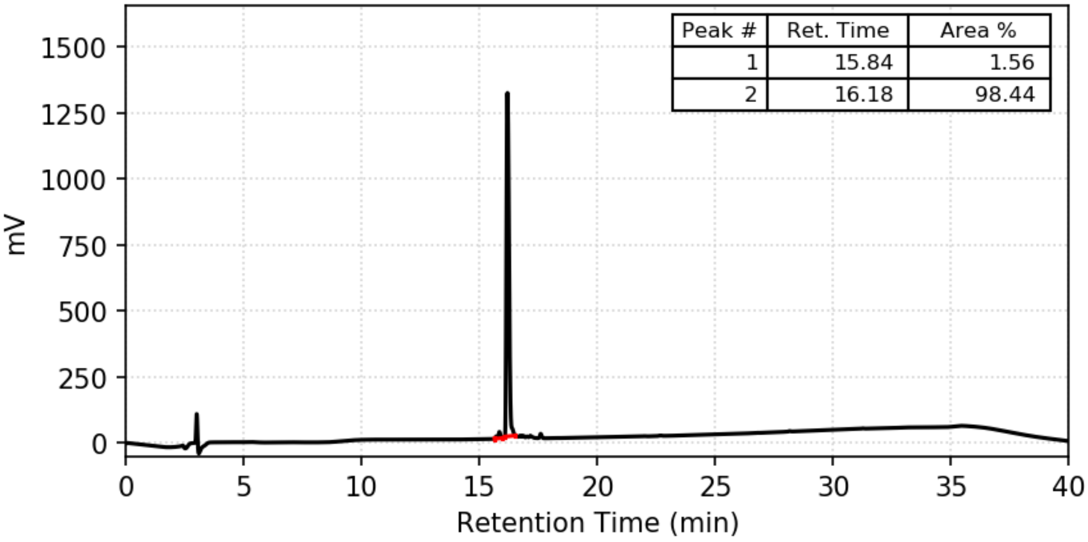

CSP1-L4NV

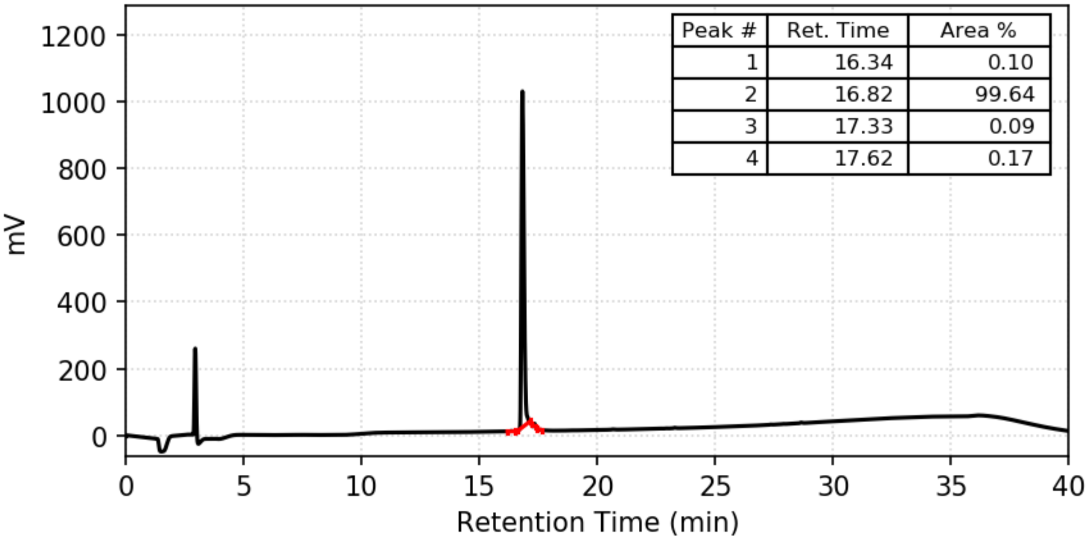

CSP1-L4V

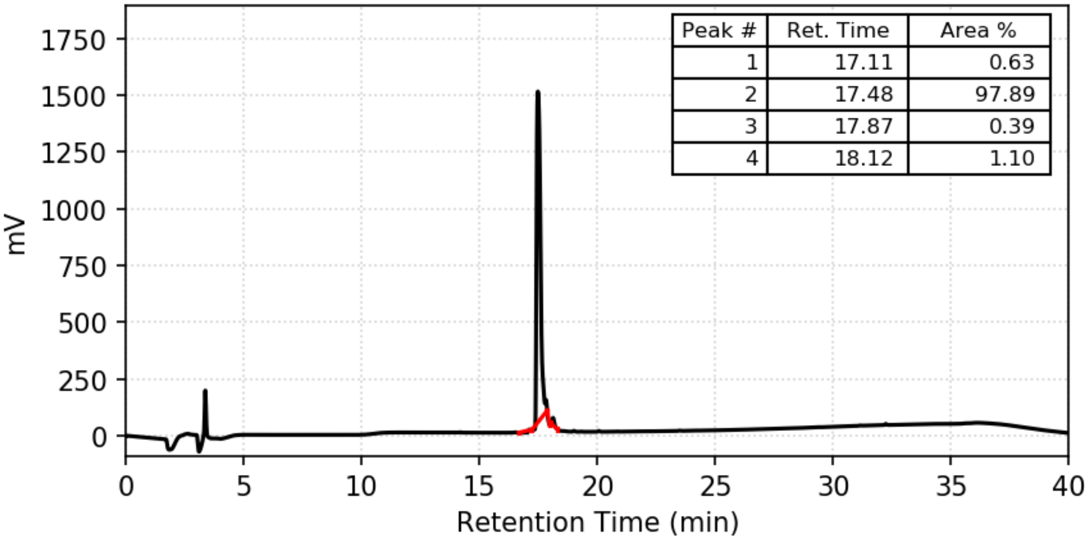

CSP1-F7FG

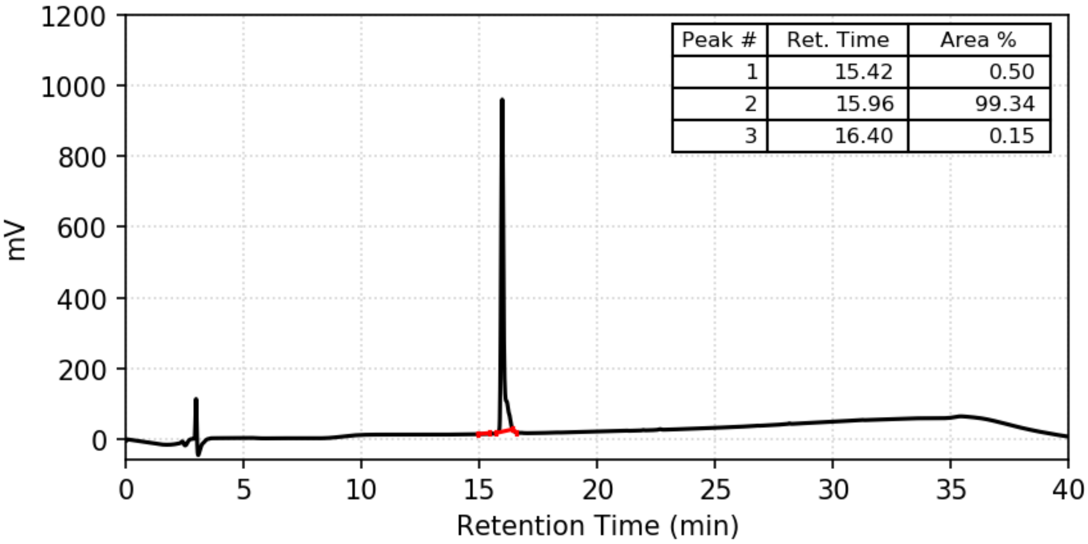

CSP1-F7HF

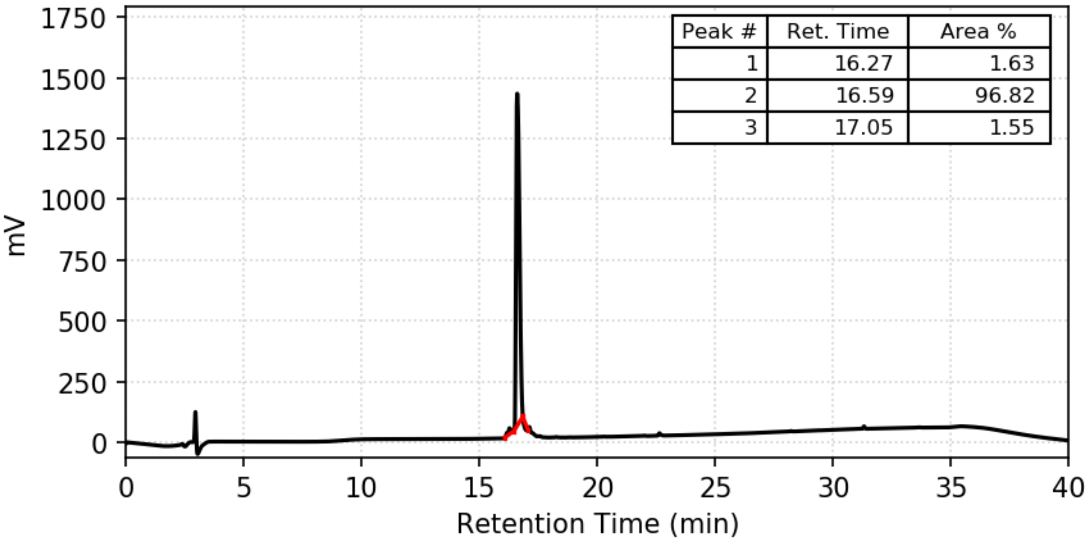

CSP1-F7Y

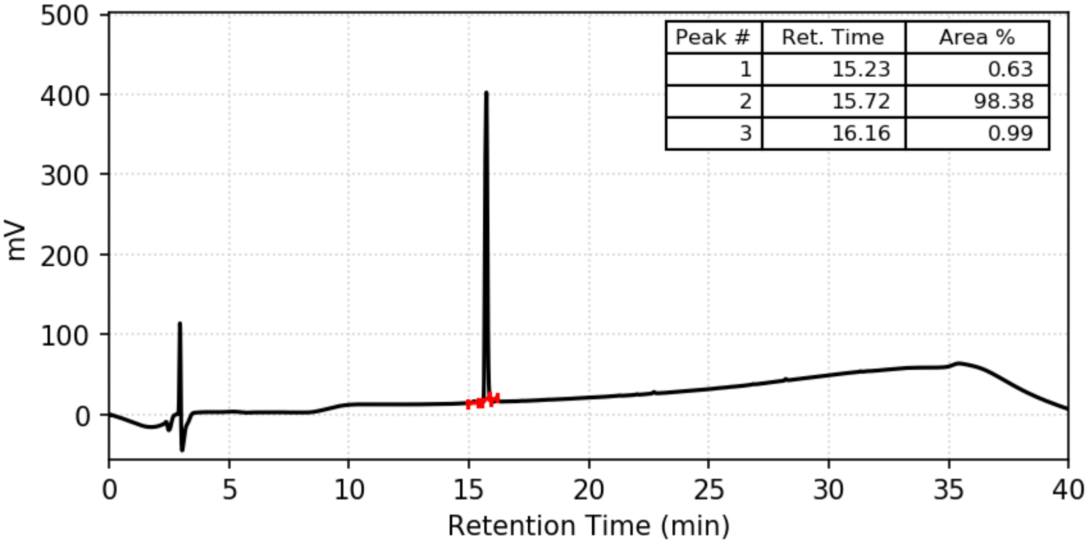

CSP1-F8FG

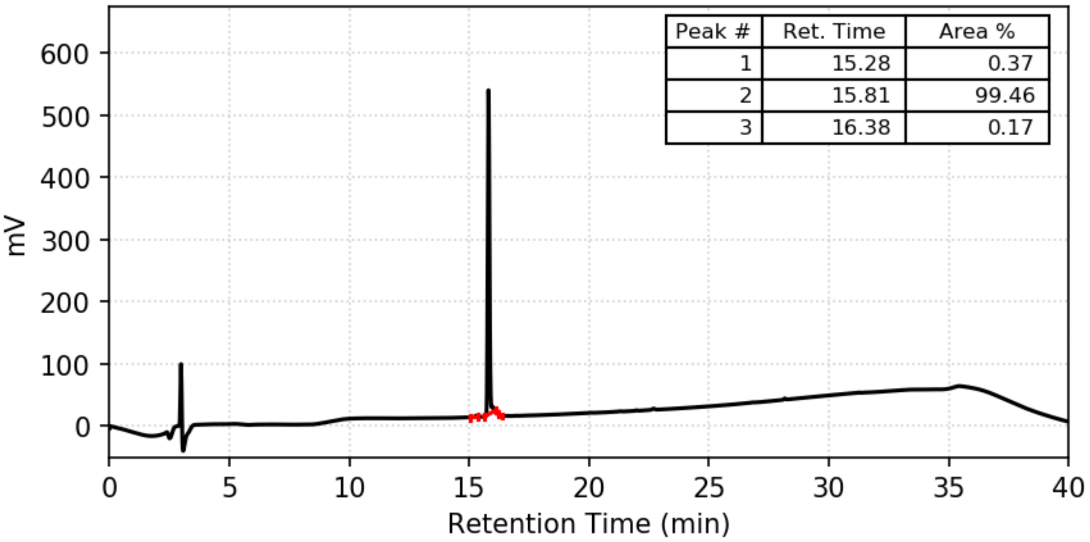

CSP1-F8HF

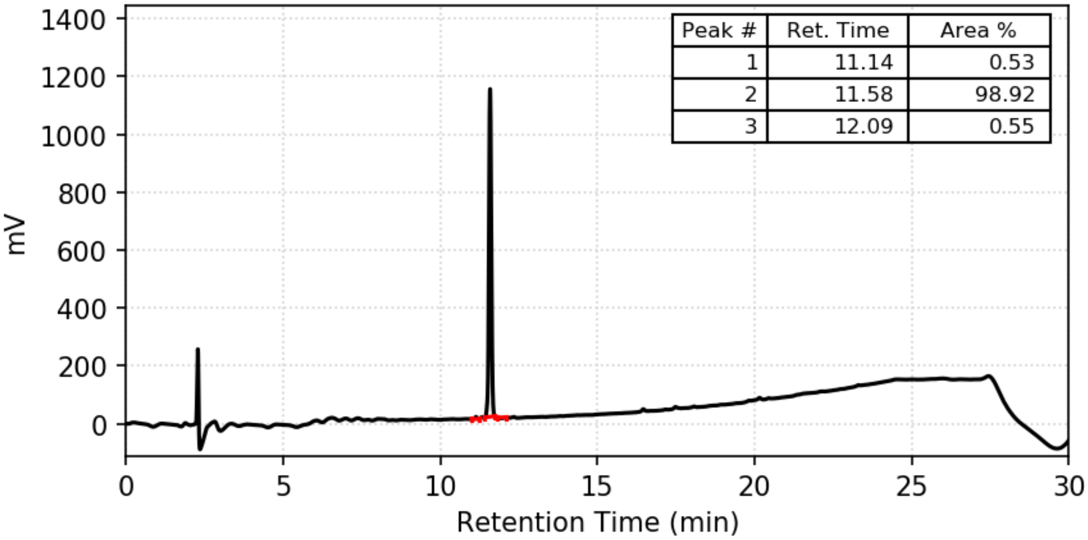

CSP1-F8Y

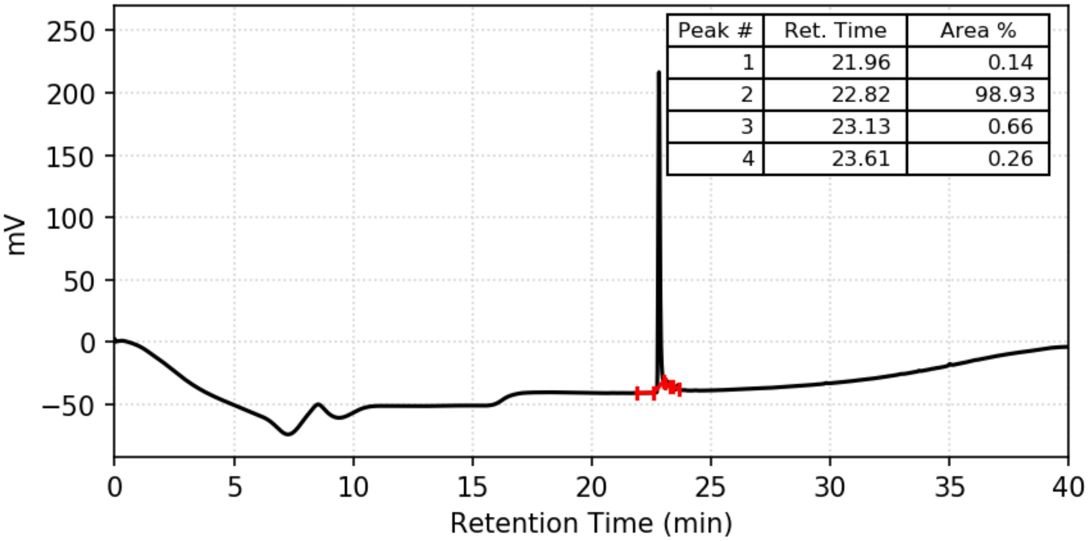

CSP1-F11FG

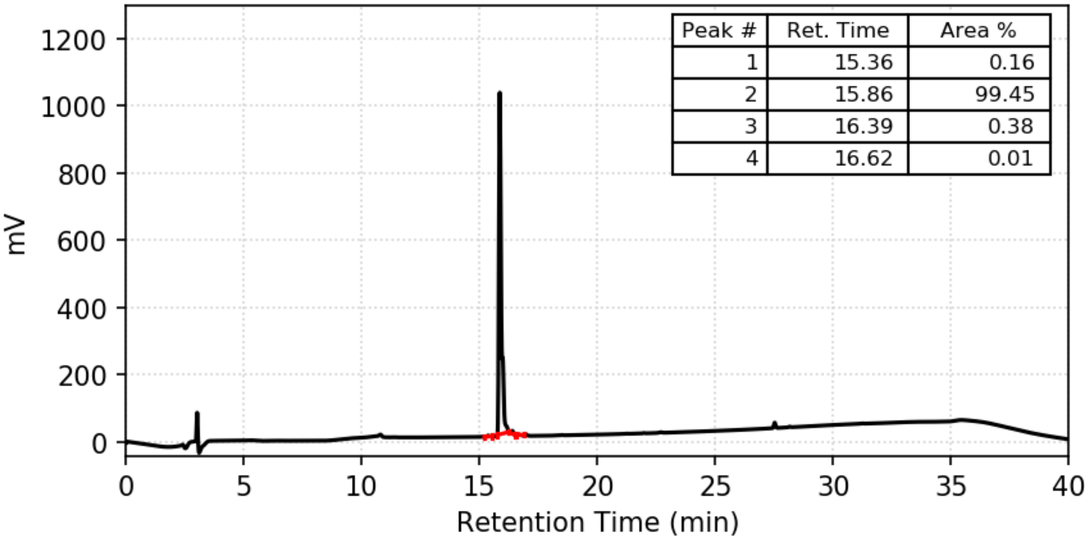

CSP1-F11HF

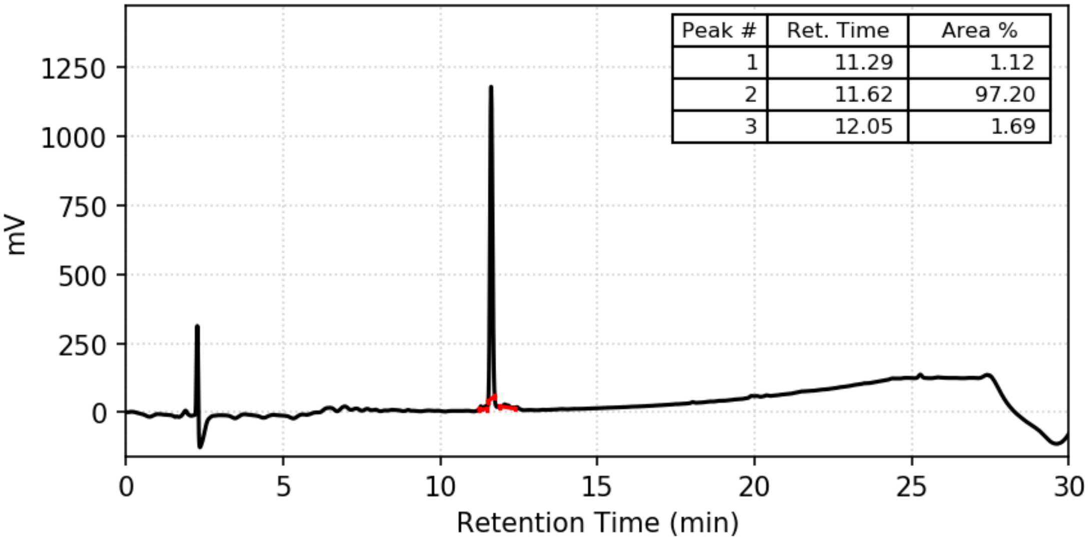

CSP1-F11Y

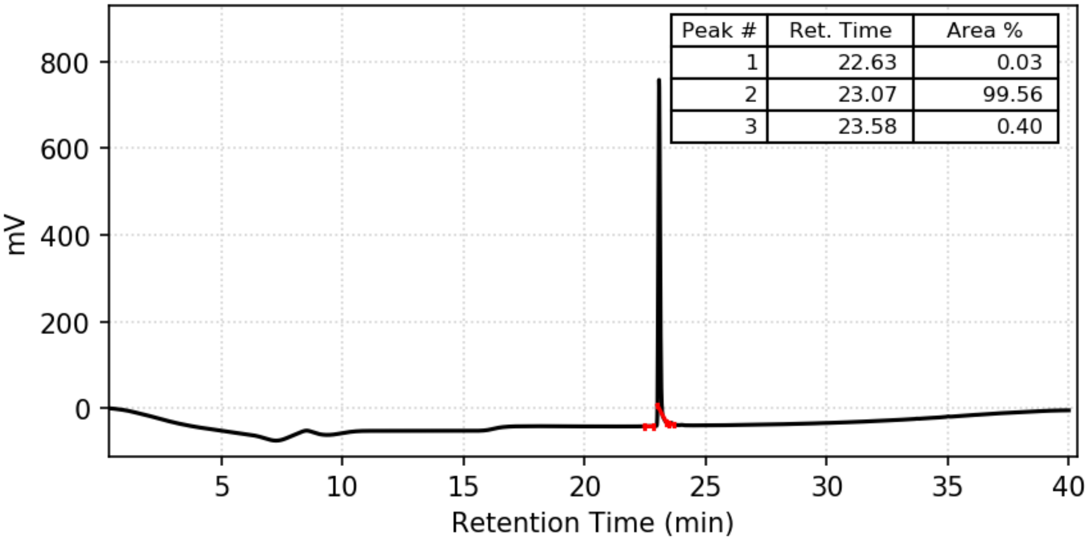

CSP1-I12L

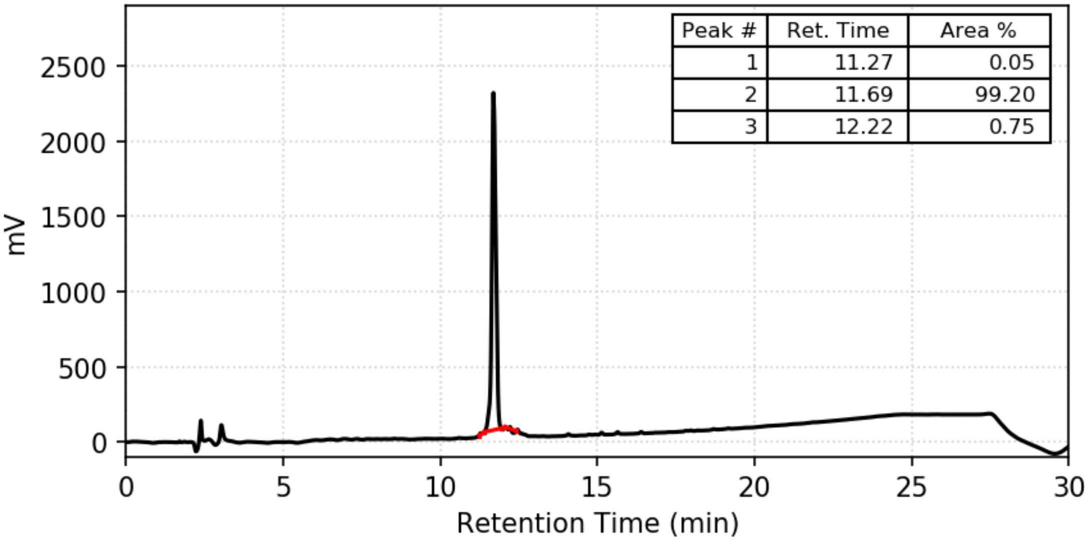

CSP1-I12NL

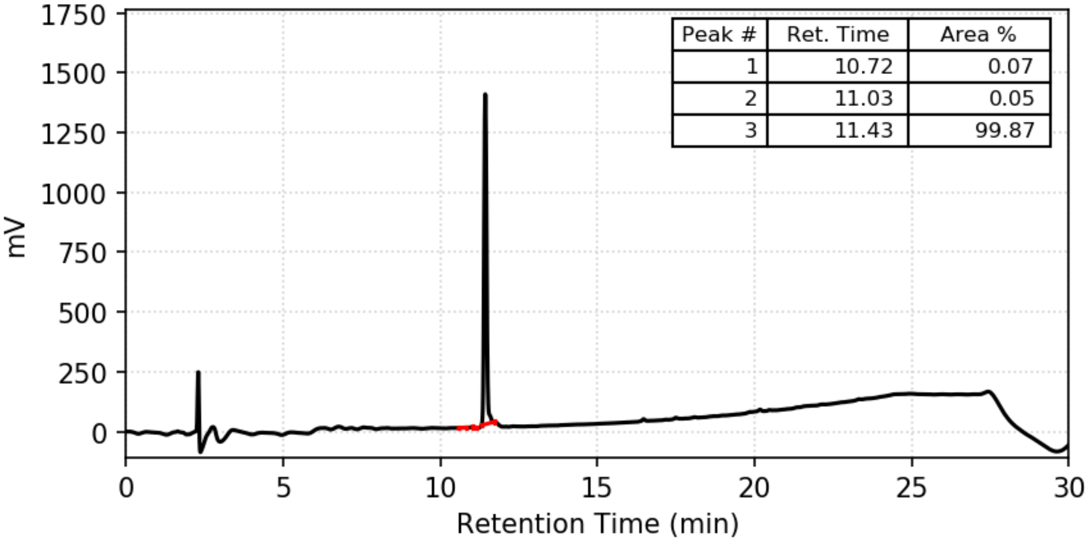

CSP1-I12NV

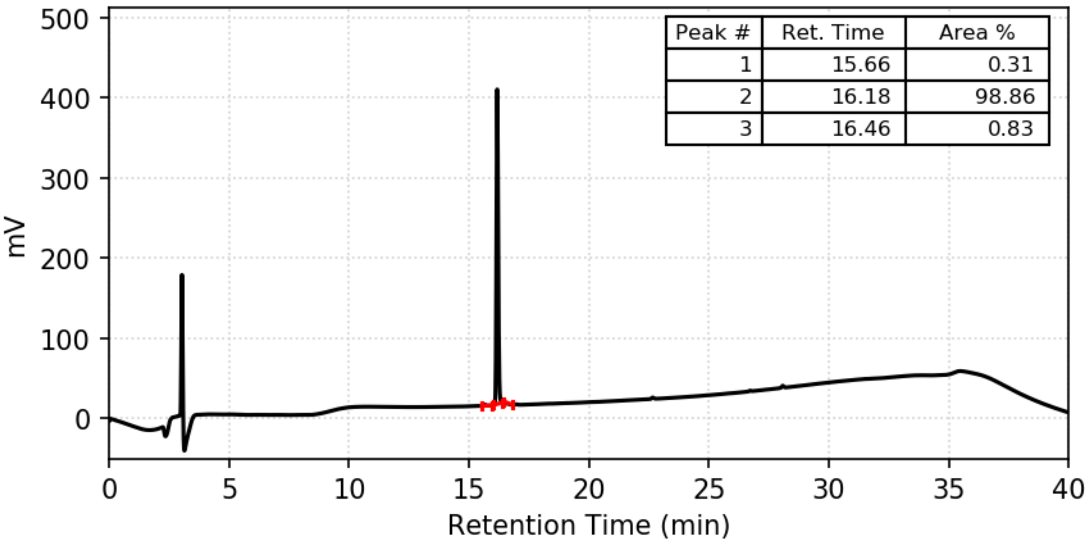

CSP1-I12V

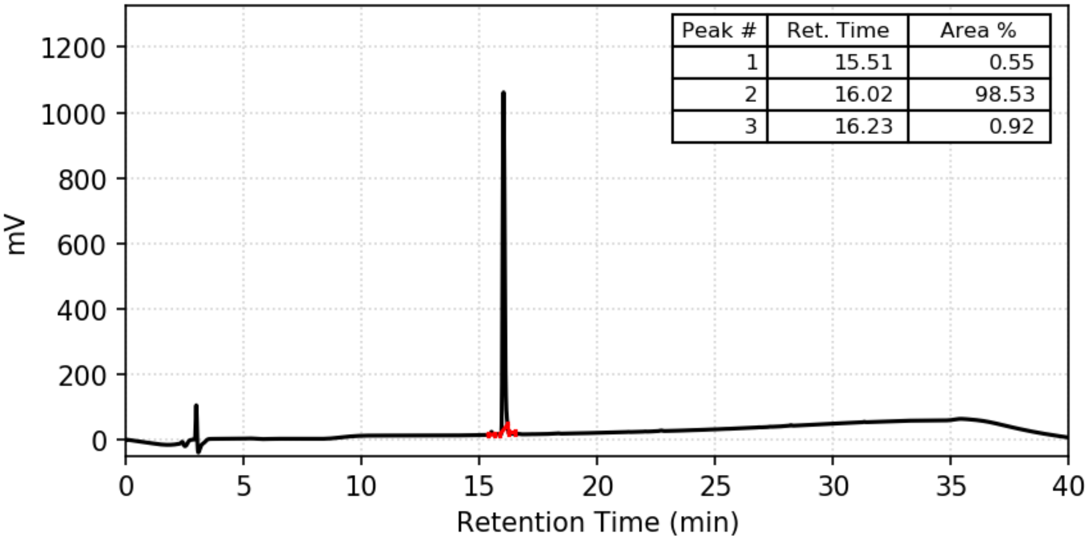

CSP1-L13I

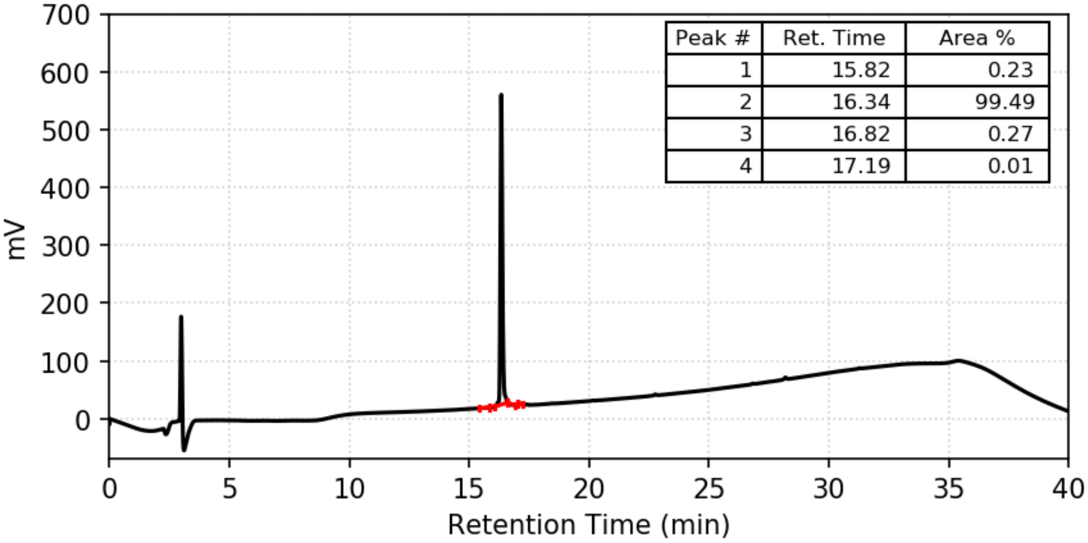

CSP1-L13NL

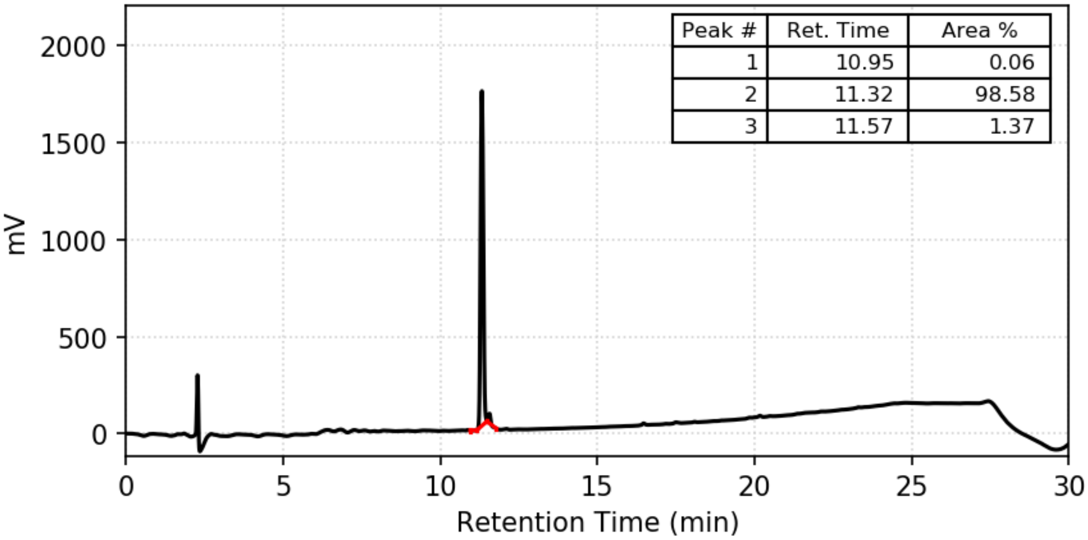

CSP1-L13NV

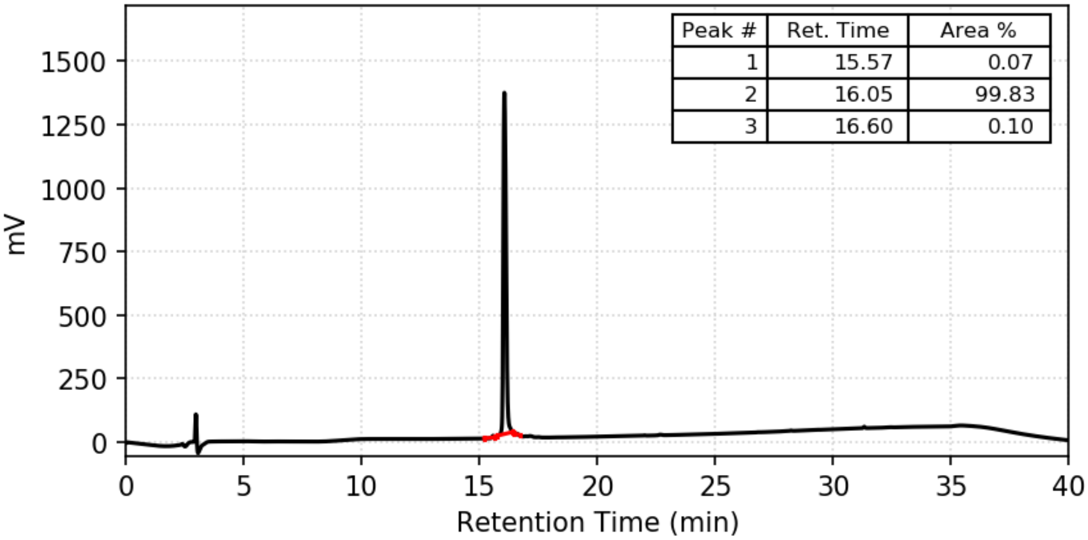

CSP1-L13V

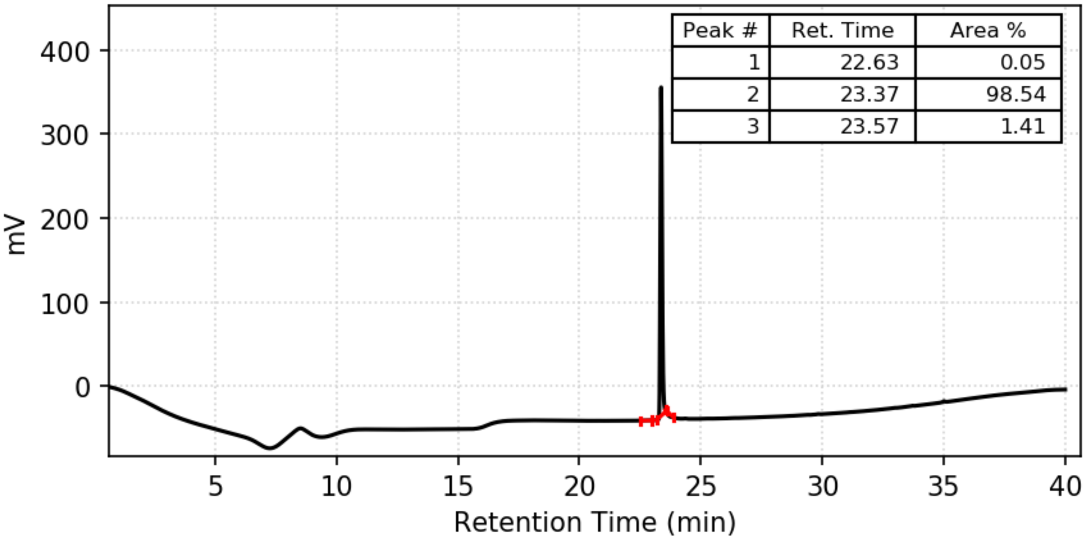
Peak #
Ret. Time
Area %
1
22.63
0.05
2
23.37
98.54
3
23.57
1.41
400
300
200
100
0
mV
5
10
15
20
25
30
35
40
Retention Time (min)

MS and HPLC data for CSP1 analogs

Table S1. MS and HPLC data for CSP1 analogs.

Compound Name	Calc. EM MH_3^{3+}	Obs. EM MH_3^{3+}	Purity (%)
CSP1-L4I	1122.1371	1122.1354	>99
CSP1-L4NL	1122.1371	1122.1338	>98
CSP1-L4NV	1115.1293	1115.1337	>99
CSP1-L4V	1115.1293	1115.1292	>97
CSP1-F7FG	1115.1293	1115.1348	>99
CSP1-F7HF	1129.1449	1129.1470	>96
CSP1-F7Y	1130.1346	1130.1401	>98
CSP1-F8FG	1115.1293	1115.1254	>99
CSP1-F8HF	1129.1449	1129.1397	>98
CSP1-F8Y	1130.1346	1130.1310	>98
CSP1-F11FG	1115.1293	1115.1247	>99
CSP1-F11HF	1129.1449	1129.1430	>97
CSP1-F11Y	1130.1346	1130.1315	>99
CSP1-I12L	1122.1371	1122.1352	>99
CSP1-I12NL	1122.1371	1122.1338	>99
CSP1-I12NV	1115.1293	1115.1291	>98
CSP1-I12V	1115.1293	1115.1260	>98
CSP1-L13I	1122.1371	1122.1352	>99
CSP1-L13NL	1122.1371	1122.1416	>98
CSP1-L13NV	1115.1293	1115.1349	>99
CSP1-L13V	1115.1293	1115.1247	>98

Bioassay initial screening results

S. pneumoniae **D39pcomX::lacZ (ComD1)**

Agonism assays were performed at 10 µM concentration. CSP1 was used as the positive control (100%) while DMSO was used as the negative control (0%). Percent (%) *comX* activation was measured by normalizing the Miller units obtained for each peptide to that of CSP1. All peptides were screened in triplicate over three separate trials. Error bars indicate standard error of the mean of nine values.

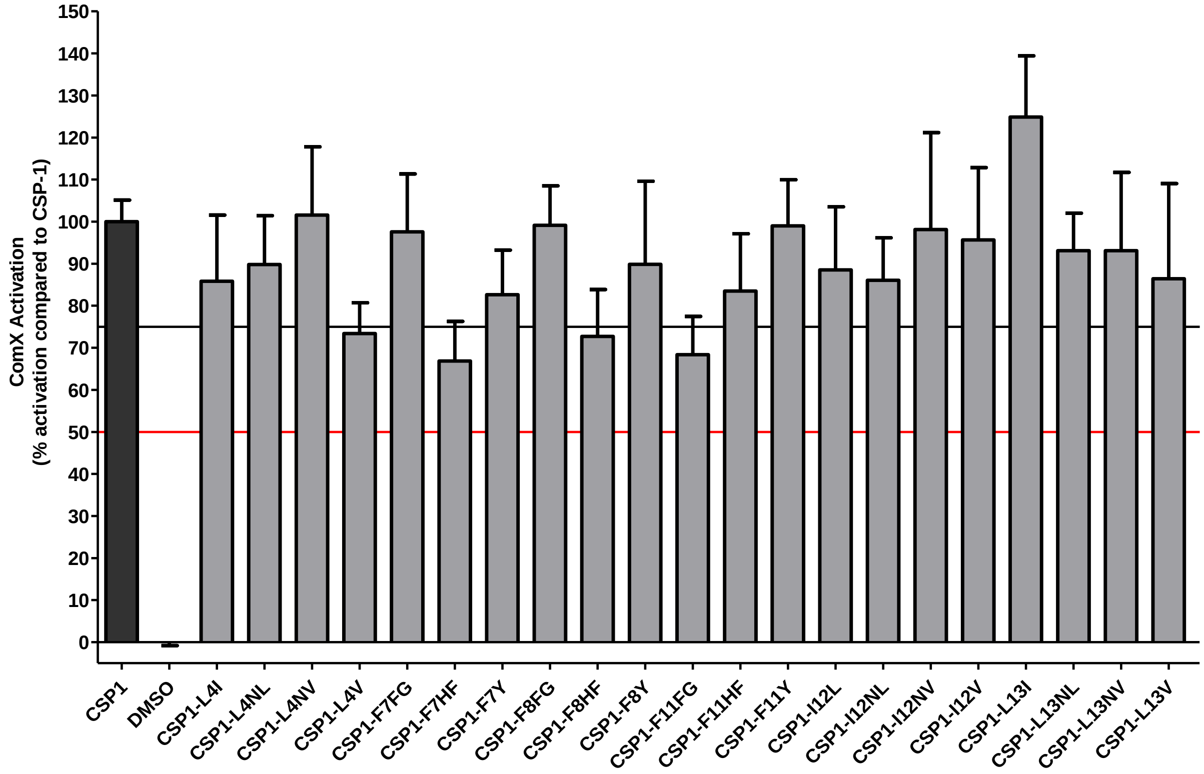

Figure S1. Primary agonism screening assay data for the CSP1 analogs. Peptides that exhibited over 75% activation were further evaluated to determine their EC_{50}.

***S. pneumoniae* TIGR4pcomX::lacZ (ComD2)**

Agonism assays were performed at 10 µM concentration. CSP2 was used as the positive control (100%) while DMSO was used as the negative control (0%). Percent (%) *comX* activation was measured by normalizing the Miller units obtained for each peptide to that of CSP2. All peptides were screened in triplicate over three separate trials. Error bars indicate standard error of the mean of nine values.

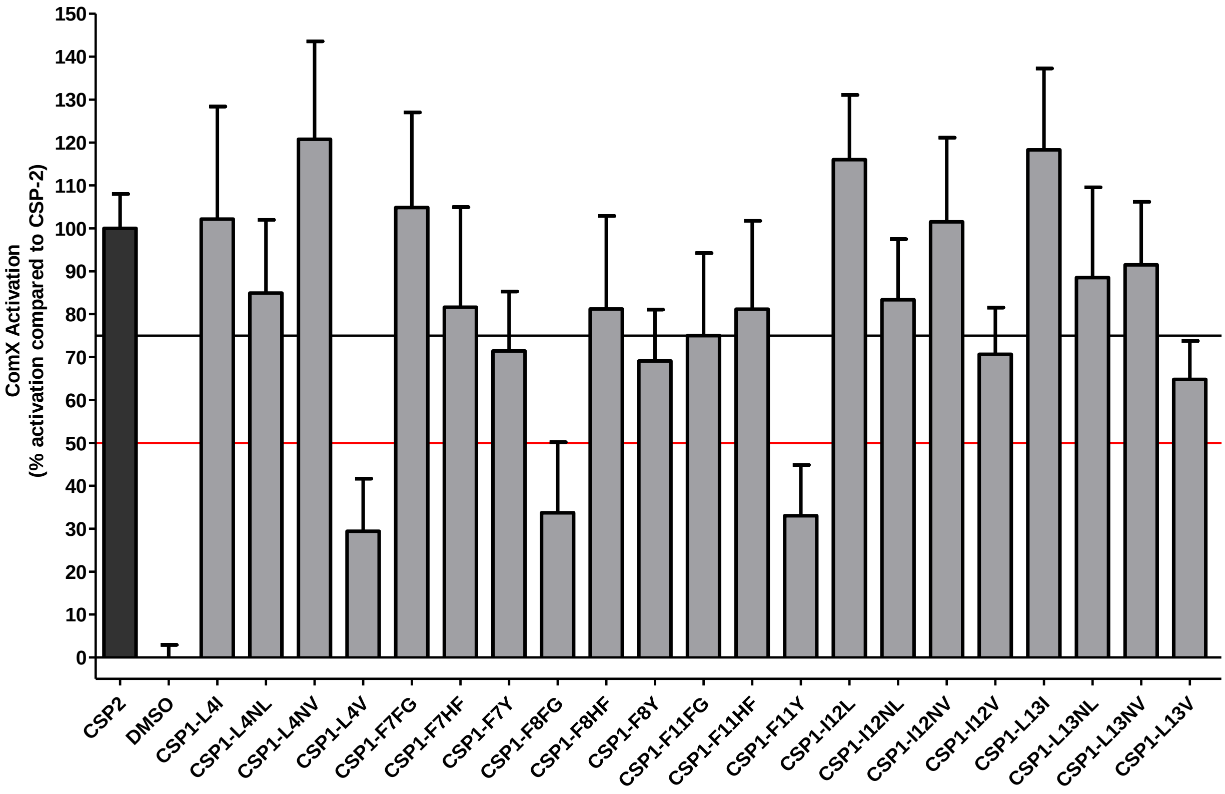

Figure S2. Primary agonism screening assay data for the CSP1 analogs. Peptides that exhibited over 75% activation were further evaluated to determine their EC_{50} while peptides that exhibited less than 50% activation were evaluated as potential competitive inhibitors.

Antagonism assays were performed at 10 µM concentration of peptides against 250 nM concentration of CSP2. CSP2 (250 nM) was used as the positive control (100%) while DMSO was used as the negative control (0%). Percent (%) *comX* activation was measured by normalizing the Miller units obtained for each peptide to that of CSP2. All peptides were screened in triplicate over three separate trials. Error bars indicate standard error of the mean of nine values.

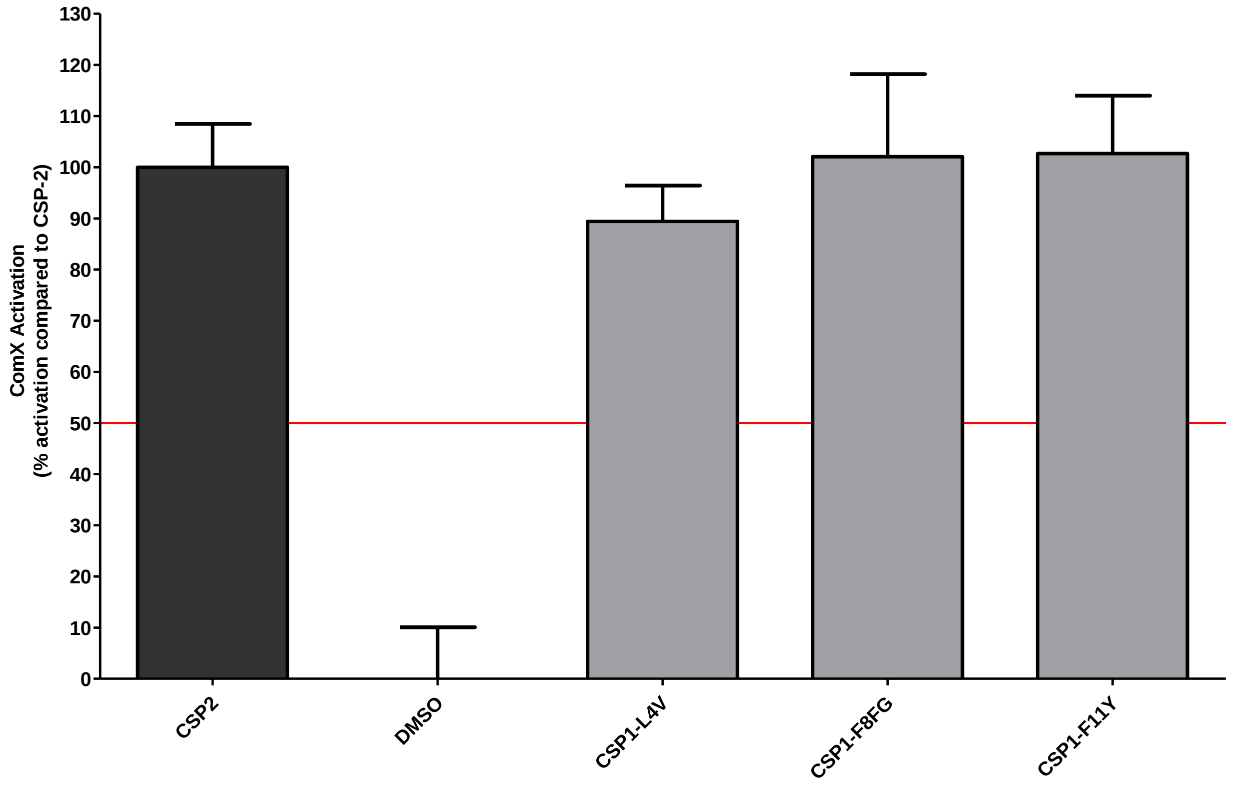

Figure S3. Primary antagonism screening assay data for the CSP1 analogs. None of the peptides exhibited less than 50% activation.

Dose Response Curves

CSP1 analogs were screened over varying concentrations in the two indicated *S. pneumoniae* beta-galactosidase reporter strains. Each dose response experiment was performed in triplicate on three separate occasions (i.e., experiments (Exp.) #1-3; shown for each peptide below). Error bars indicate standard error of the mean of triplicate values. In each plot the peptide as well as its EC_{50} value and 95% confidence interval values (95% CI) are indicated in the upper left corner.

S. pneumoniae **D39pcomX::lacZ (ComD1)**

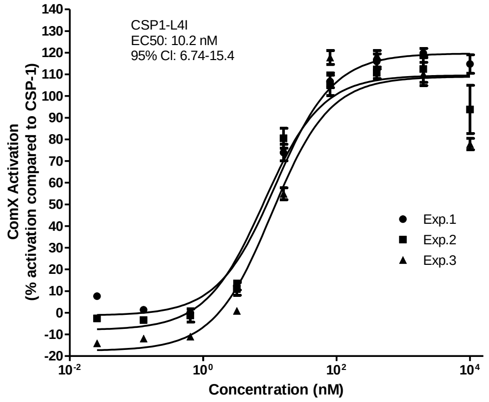

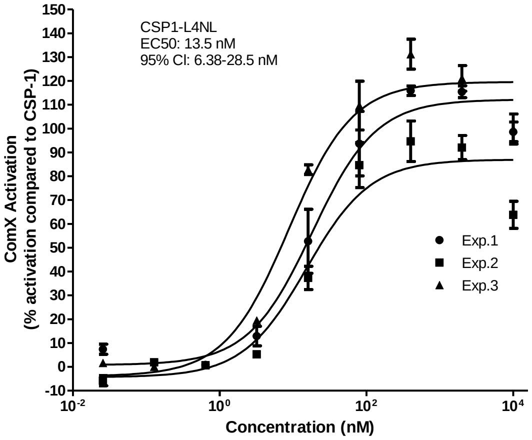

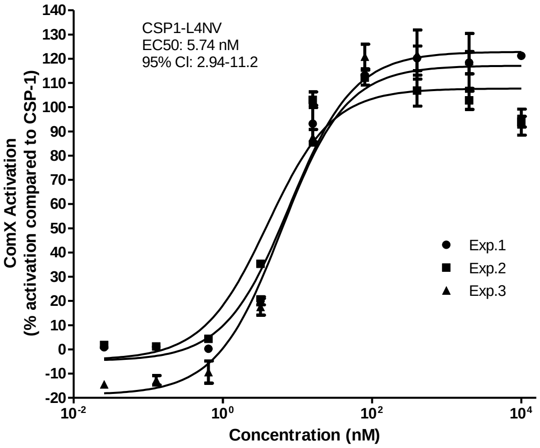

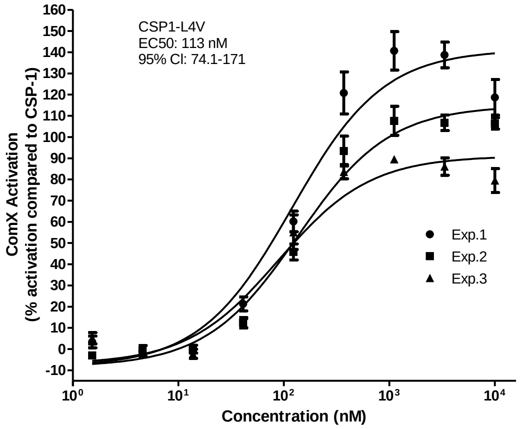

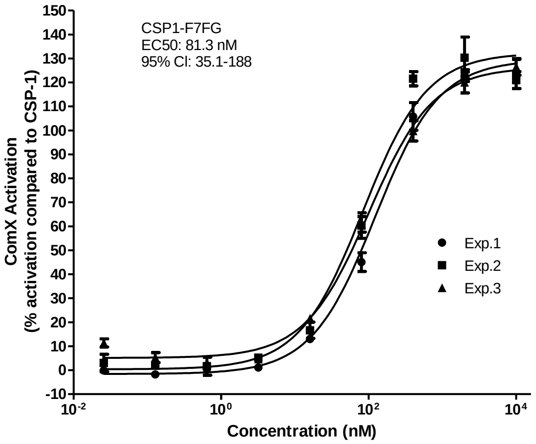

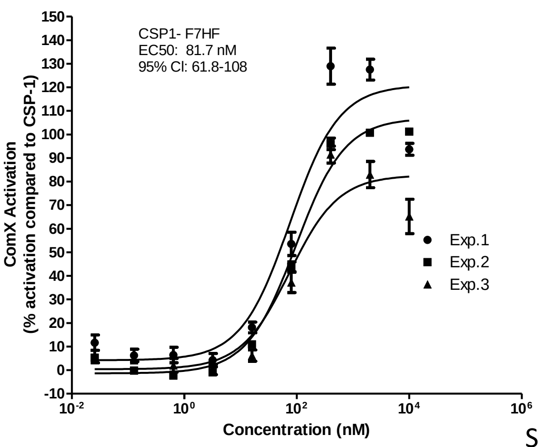

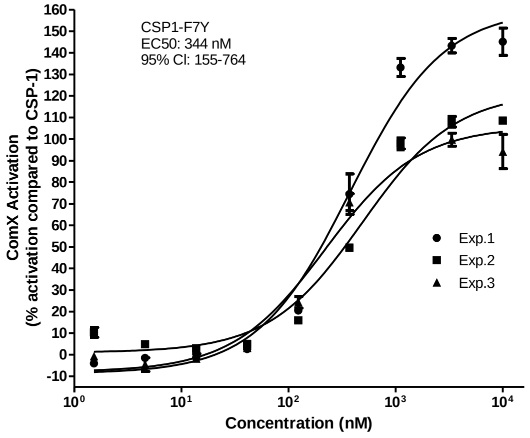
CSP1-F7Y
EC50: 344 nM
95% CI: 155-764
Exp.1
Exp.2
Exp.3
ComX Activation
(% activation compared to CSP-1)
Concentration (nM)

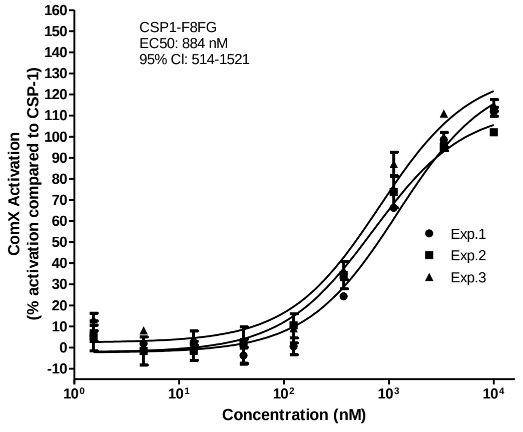
CSP1-F8FG
EC50: 884 nM
95% CI: 514-1521
Exp.1
Exp.2
Exp.3
ComX Activation
(% activation compared to CSP-1)
Concentration (nM)

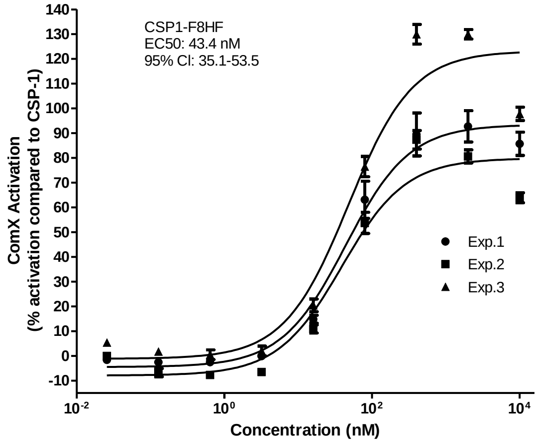
CSP1-F8HF
EC50: 43.4 nM
95% CI: 35.1-53.5
Exp.1
Exp.2
Exp.3
ComX Activation
(% activation compared to CSP-1)
Concentration (nM)

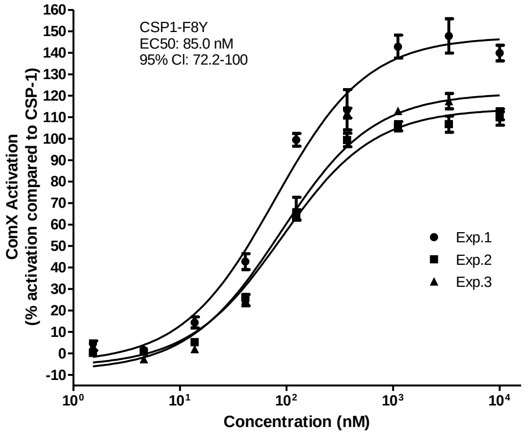
CSP1-F8Y
EC50: 85.0 nM
95% CI: 72.2-100
Exp.1
Exp.2
Exp.3
ComX Activation
(% activation compared to CSP-1)
Concentration (nM)

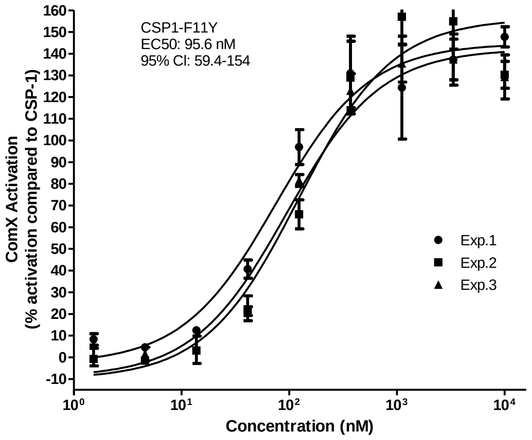
CSP1-F11Y
EC50: 95.6 nM
95% CI: 59.4-154
Exp.1
Exp.2
Exp.3
ComX Activation
(% activation compared to CSP-1)
Concentration (nM)

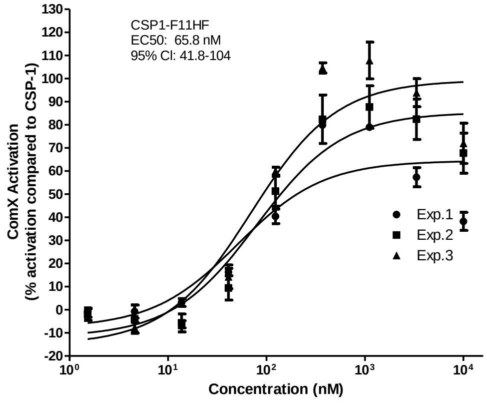
CSP1-F11HF
EC50: 65.8 nM
95% CI: 41.8-104
Exp.1
Exp.2
Exp.3
ComX Activation
(% activation compared to CSP-1)
Concentration (nM)

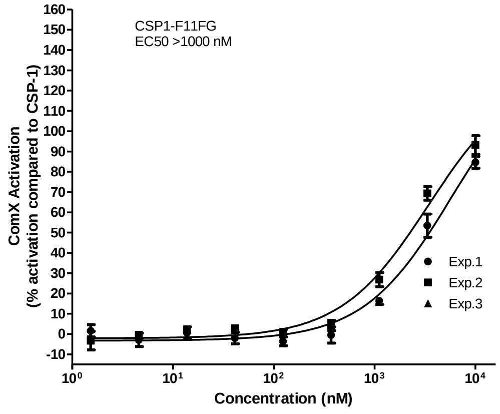
CSP1-F11FG
EC50 >1000 nM
Exp.1
Exp.2
Exp.3
ComX Activation
(% activation compared to CSP-1)
Concentration (nM)

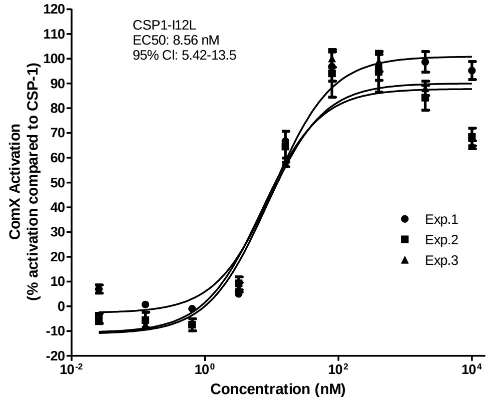
CSP1-I12L
EC50: 8.56 nM
95% CI: 5.42-13.5
Exp.1
Exp.2
Exp.3
ComX Activation
(% activation compared to CSP-1)
Concentration (nM)

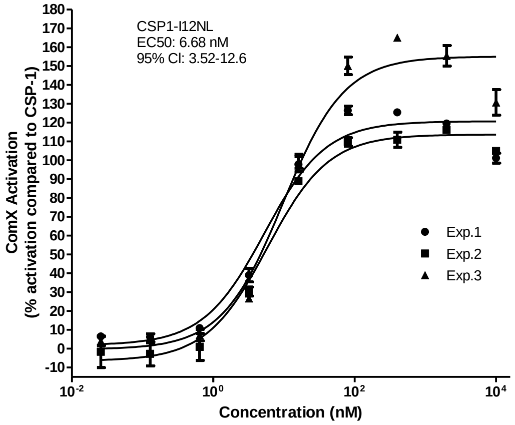
CSP1-I12NL
EC50: 6.68 nM
95% CI: 3.52-12.6
Exp.1
Exp.2
Exp.3
ComX Activation
(% activation compared to CSP-1)
Concentration (nM)

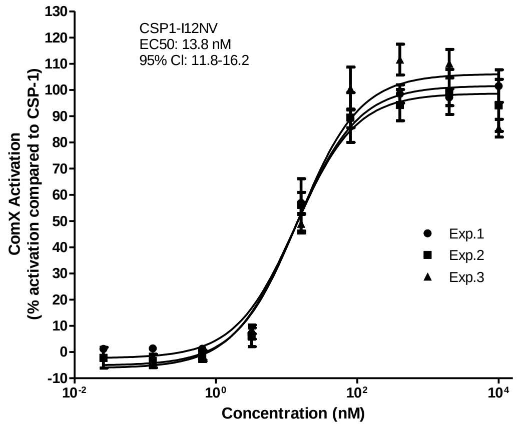
CSP1-I12NV
EC50: 13.8 nM
95% CI: 11.8-16.2
Exp.1
Exp.2
Exp.3
ComX Activation
(% activation compared to CSP-1)
Concentration (nM)

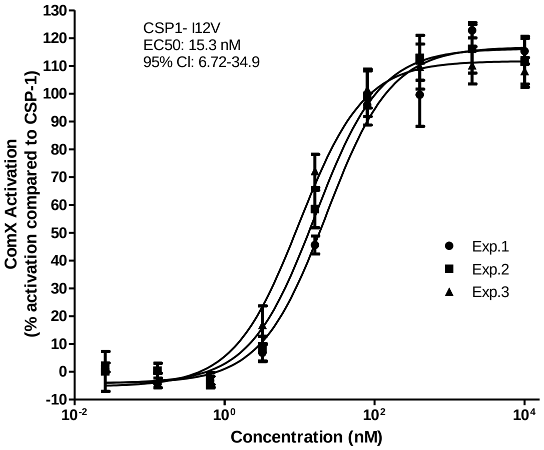
CSP1- I12V
EC50: 15.3 nM
95% CI: 6.72-34.9
Exp.1
Exp.2
Exp.3
ComX Activation
(% activation compared to CSP-1)
Concentration (nM)

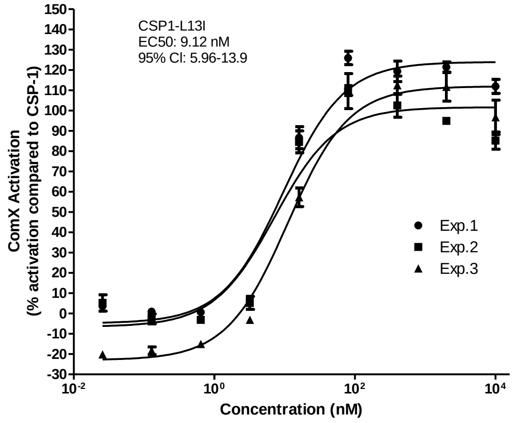
CSP1-L13I
EC50: 9.12 nM
95% CI: 5.96-13.9
Exp.1
Exp.2
Exp.3
ComX Activation
(% activation compared to CSP-1)
Concentration (nM)

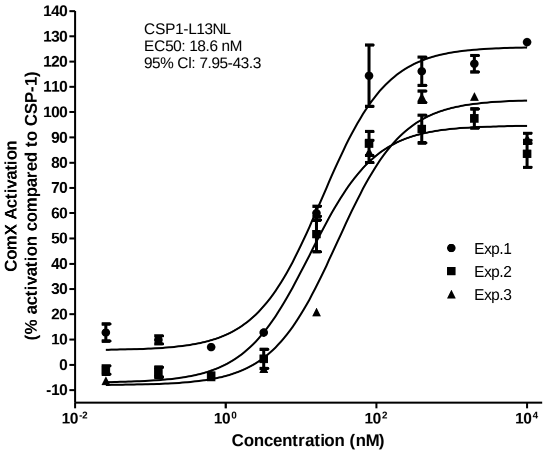
CSP1-L13NL
EC50: 18.6 nM
95% CI: 7.95-43.3
Exp.1
Exp.2
Exp.3
ComX Activation
(% activation compared to CSP-1)
Concentration (nM)

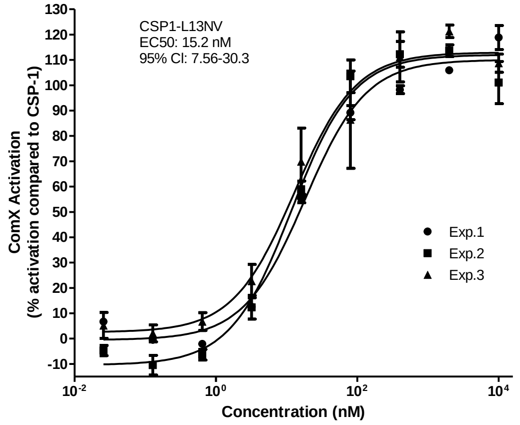
CSP1-L13NV
EC50: 15.2 nM
95% CI: 7.56-30.3
Exp.1
Exp.2
Exp.3
ComX Activation
(% activation compared to CSP-1)
Concentration (nM)

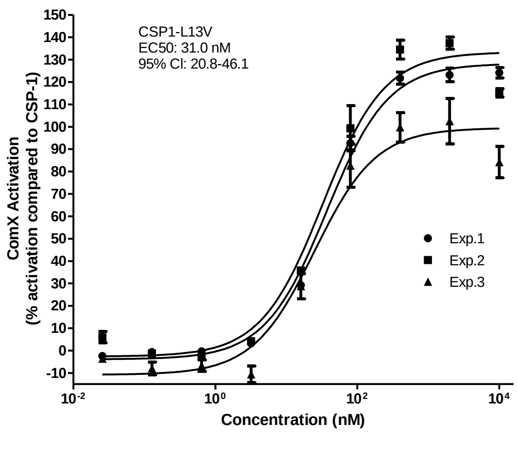
CSP1-L13V
EC50: 31.0 nM
95% CI: 20.8-46.1
Exp.1
Exp.2
Exp.3
ComX Activation
(% activation compared to CSP-1)
Concentration (nM)

S. pneumoniae TIGR4pcomX::lacZ (ComD2)

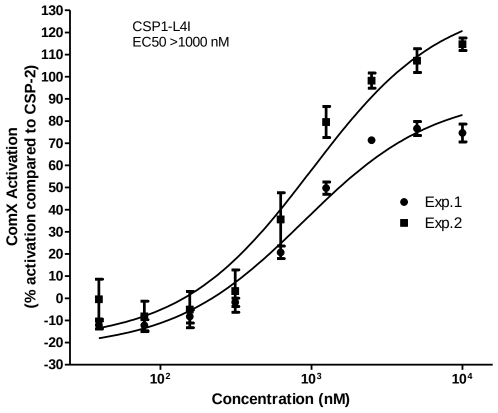

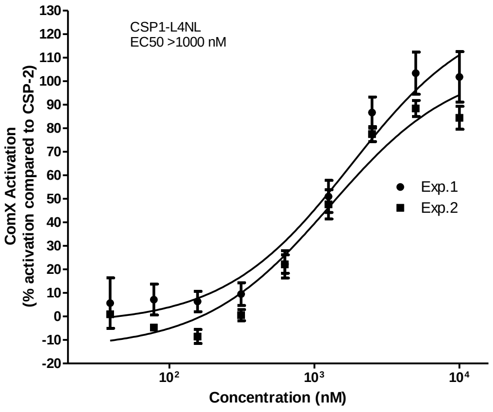

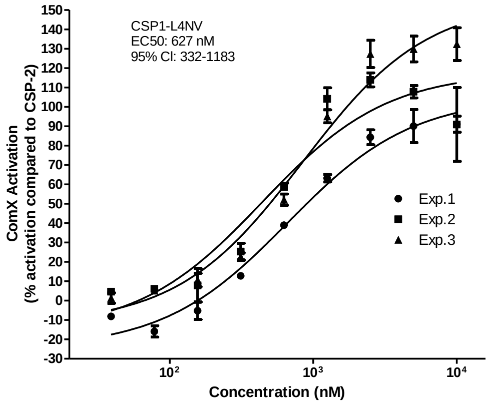

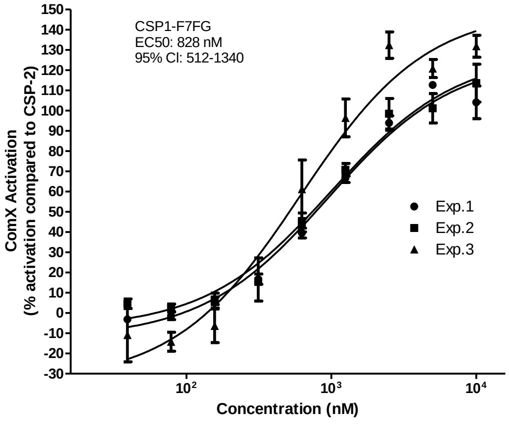

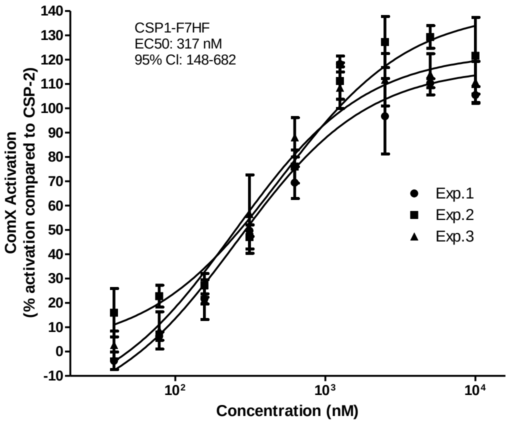

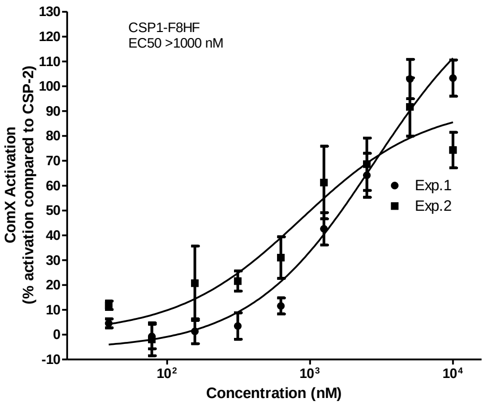

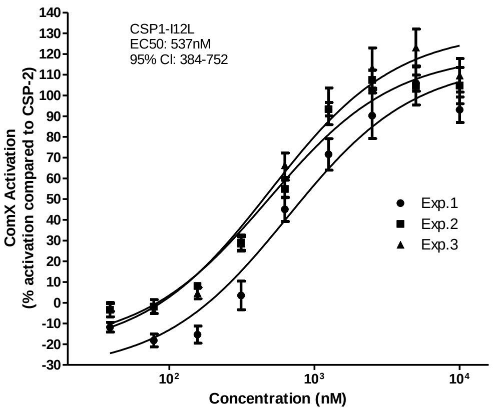

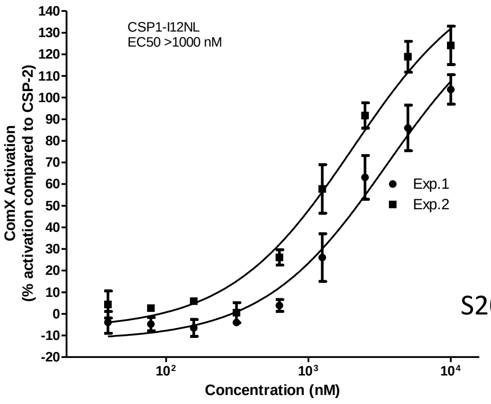

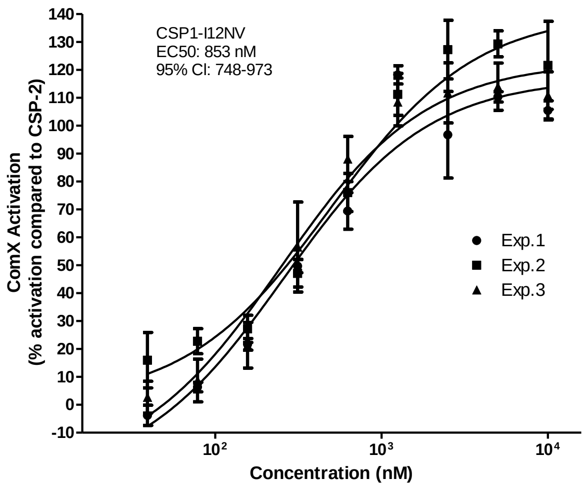
CSP1-I12NV
EC50: 853 nM
95% CI: 748-973
ComX Activation
(% activation compared to CSP-2)
Concentration (nM)
Exp.1
Exp.2
Exp.3

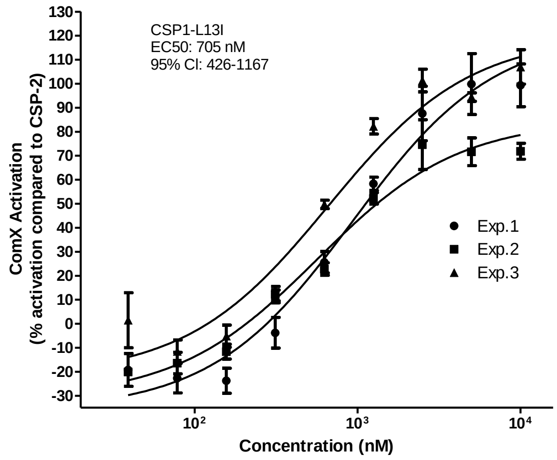
CSP1-L13I
EC50: 705 nM
95% CI: 426-1167
ComX Activation
(% activation compared to CSP-2)
Concentration (nM)
Exp.1
Exp.2
Exp.3

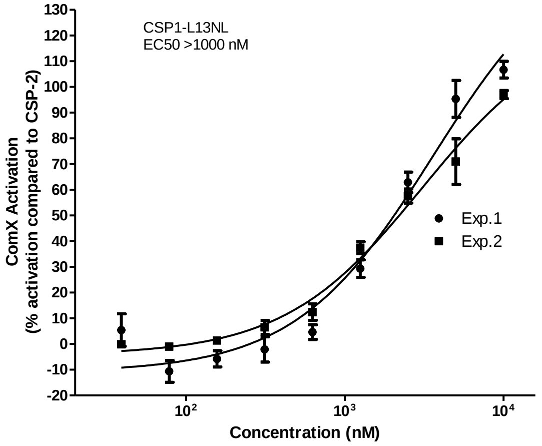
CSP1-L13NL
EC50 >1000 nM
ComX Activation
(% activation compared to CSP-2)
Concentration (nM)
Exp.1
Exp.2

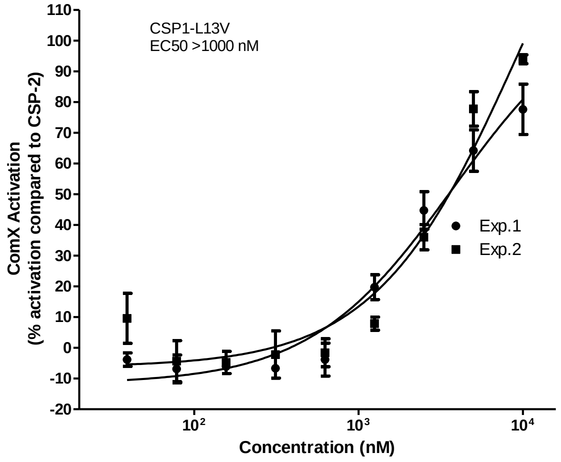
CSP1-L13V
EC50 >1000 nM
ComX Activation
(% activation compared to CSP-2)
Concentration (nM)
Exp.1
Exp.2

Circular dichroism (CD) spectra

Table S2. ComD1 EC_{50} values, % Helicity and estimated secondary structure contents (in 20% TFE) data for CSP1 analogs.

Peptide Name	Helicity (%)[a]	EC_{50} ComD1 (nM)	Estimated secondary structure contents (%)[b]				
			α-helix	Antiparallel *β*-sheet	Parallel *β*-sheet	*β*-turn	Others
CSP1	20.1%	10.3	32.3	5.4	0.0	8.70	53.7
CSP1-L4I	34.8%	10.2	37.0	0.0	0.0	13.0	50.1
CSP1-L4NL	29.2%	13.5	30.8	0.0	0.0	13.8	55.4
CSP1-L4NV	34.0%	5.74	36.0	0.0	0.0	13.3	50.7
CSP1-L4V	30.1%	113	30.9	0.0	0.0	13.9	55.2
CSP1-F7FG	26.3%	81.3	25.4	3.0	0.0	14.1	57.5
CSP1-F7HF	26.7%	81.7	26.1	1.9	0.0	14.7	57.3
CSP1-F7Y	31.8%	344	36.6	0.0	0.0	12.3	51.1
CSP1-F8FG	13.4%	884	6.2	5.4	1.6	17.2	69.6
CSP1-F8HF	19.4%	43.4	15.3	3.5	0.0	16.3	64.9
CSP1-F8Y	25.7%	85.0	30.8	0.0	0.0	14.1	55.1
CSP1-F11FG	15.9%	>1000	10.2	2.8	0.5	16.5	70.0
CSP1-F11HF	32.3%	65.8	35.1	0.0	0.0	14.3	50.6
CSP1-F11Y	29.2%	95.6	34.2	0.0	0.0	14.0	51.9
CSP1-I12L	34.1%	8.56	35.6	0.0	0.0	13.6	50.8
CSP1-I12NL	24.7%	6.68	26.1	0.0	0.0	15.3	58.6
CSP1-I12NV	25.6%	13.8	24.2	0.0	0.0	14.7	61.1
CSP1-I12V	29.0%	15.3	32.2	0.0	0.0	14.7	53.1
CSP1-L13I	27.7%	9.12	27.6	0.0	0.0	14.1	58.3
CSP1-L13NL	17.3%	18.6	12.4	2.7	0.0	16.4	68.4
CSP1-L13NV	30.9%	15.2	33.4	0.0	0.0	14.0	52.6
CSP1-L13V	25.8%	31.0	27.9	0.0	0.0	15.1	57.0

[a] Percent (%) helicity was calculated using the mean residue ellipticity at 222 nm.[1] [b] Secondary structure contents were calculated using BeStSel (Beta Structure Selection) method.[2]

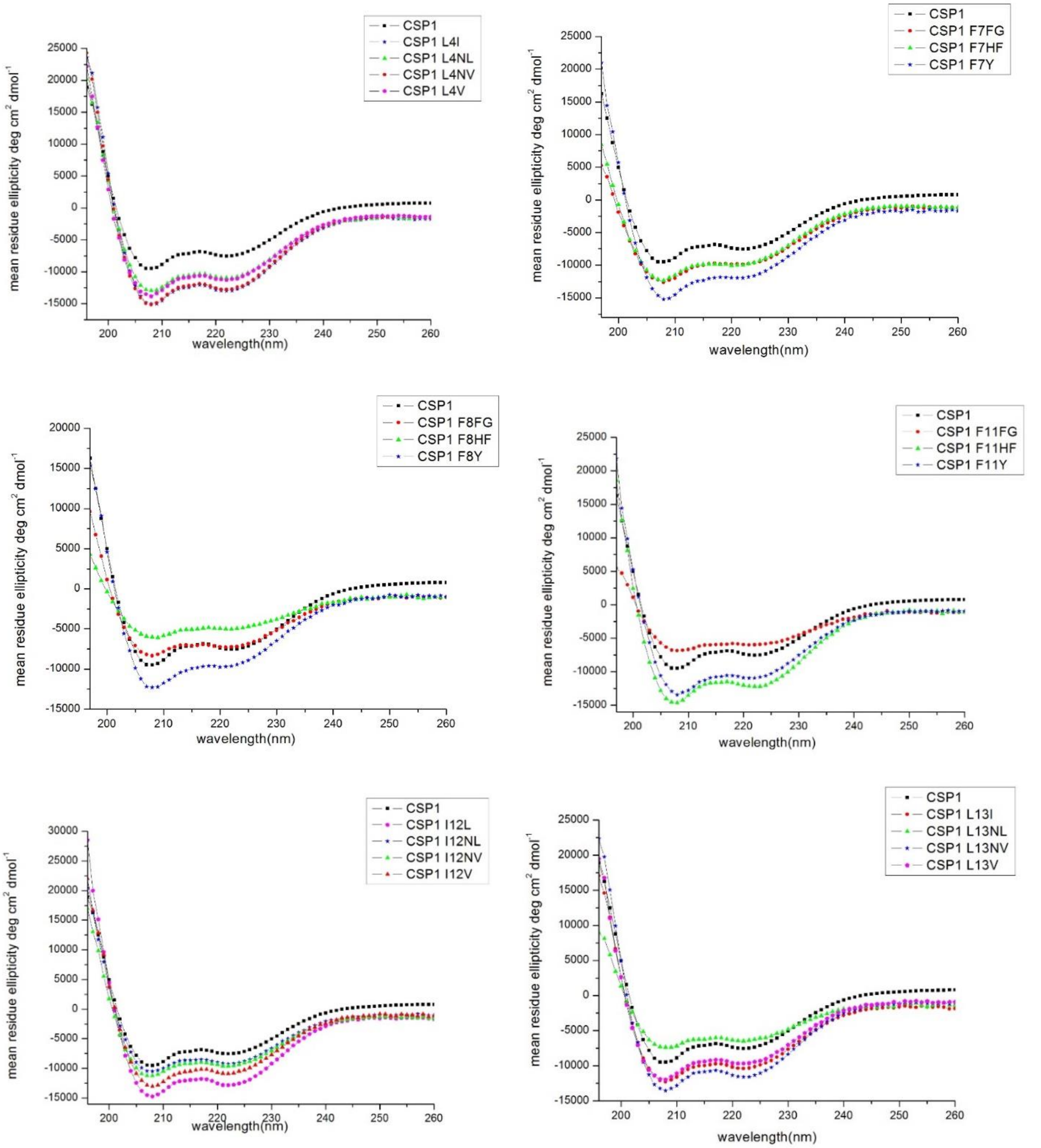

Figure S4. CD spectra of the CSP1 analogs in membrane mimicking conditions (20% TFE: 80% PBS, pH 7.4). All the measurements were performed with a peptide concentration of 200 µM. CSP1 was added as a control. The peptides exhibited varying degrees of an α-helix pattern.

References

1. Luo, P.; Baldwin, R. L., Mechanism of helix induction by trifluoroethanol: a framework for extrapolating the helix-forming properties of peptides from trifluoroethanol/water mixtures back to water. *Biochemistry* **1997,** *36* (27), 8413-8421.

2. Micsonai, A.; Wien, F.; Kernya, L.; Lee, Y. H.; Goto, Y.; Réfrégiers, M.; Kardos, J., Accurate secondary structure prediction and fold recognition for circular dichroism spectroscopy. *Proc Natl Acad Sci U S A* **2015,** *112* (24), E3095-103.
